# Transient quantum beatings of trions in hybrid organic tri-iodine perovskite single crystal

**DOI:** 10.1038/s41467-022-29053-6

**Published:** 2022-03-17

**Authors:** Uyen N. Huynh, Ye Liu, Ashish Chanana, Dipak R. Khanal, Peter C. Sercel, Jinsong Huang, Z. Valy Vardeny

**Affiliations:** 1 Department of Physics and Astronomy, University of Utah, Salt Lake City, UT 84112 USA; 2 Department of Applied Physical Sciences, University of North Carolina at Chapel Hill, Chapel Hill, NC USA; 3 Department of Electrical Engineering, University of Utah, Salt Lake City, UT 84112 USA; 4 Center for Hybrid Organic Inorganic Semiconductors for Energy, Golden, CO 80401 USA

**Keywords:** Photonic crystals, Ultrafast lasers, Optical spectroscopy, Spintronics, Magneto-optics

## Abstract

Utilizing the spin degree of freedom of photoexcitations in hybrid organic inorganic perovskites for quantum information science applications has been recently proposed and explored. However, it is still unclear whether the stable photoexcitations in these compounds correspond to excitons, free/trapped electron-hole pairs, or charged exciton complexes such as trions. Here we investigate quantum beating oscillations in the picosecond time-resolved circularly polarized photoinduced reflection of single crystal methyl-ammonium tri-iodine perovskite (MAPbI_3_) measured at cryogenic temperatures. We observe two quantum beating oscillations (fast and slow) whose frequencies increase linearly with *B* with slopes that depend on the crystal orientation with respect to the applied magnetic field. We assign the quantum beatings to positive and negative trions whose Landé g-factors are determined by those of the electron and hole, respectively, or by the carriers left behind after trion recombination. These are ${g}_{[001]}^{e}$ = 2.52 and ${g}_{[1\bar{1}0]}^{e}\,$= 2.63 for electrons, whereas $\big|{g}_{[001]}^{h}\big|\,$= 0.28 and $\big|{g}_{[1\bar{1}0]}^{h}\big|\,$= 0.57 for holes. The obtained g-values are in excellent agreement with an 8-band K.P calculation for orthorhombic MAPbI_3_. Using the technique of resonant spin amplification of the quantum beatings we measure a relatively long spin coherence time of ~ 11 (6) nanoseconds for electrons (holes) at 4 K.

## Abstract (web summary)

Understanding photo-physics giving rise to quantum beating oscillations in hybrid organic-inorganic perovskites aids their applications in spintronics and quantum information science. Here, authors demonstrate that quantum beatings observed in single crystal perovskite at cryogenic temperatures are originating from positive and negative trions.

## Introduction

The hybrid organic–inorganic perovskite (HOIP) semiconductors have attracted intensive research interest due to their remarkable optoelectronic properties such as large absorption coefficients, strong photoluminescence emission, high carrier photogeneration efficiency and large carrier diffusion length^1–4^. Photovoltaic power-conversion efficiency of solar cells based on the HOIPs has reached 25.6%^5,6^. Moreover, the HOIPs have also been used as active materials in other optoelectronic devices, including light-emitting diodes^7,8^ and phototransistors^9^. In addition, HOIP spintronic properties have recently attracted growing attention due to the relatively long spin-relaxation time in these materials^10–12^. In particular, Rashba spin splitting^13,14^, optical spin-selection rules^15^ and magnetic-field effects on photocurrent, electroluminescence and photoluminescence in HOIP devices and films, respectively, have been observed^16^. Noteworthy potential applications of HOIPs in quantum-information science (QIS) such as quantum logic and quantum communication have been recently demonstrated^1,17,18^.

One of the key requirements for an active semiconductor in QIS is utilizing the spin degree of freedom of its lowest-lying photoexcitations. In this regard the photoexcitations in the orthorhombic HOIP phase at cryogenic temperatures are particularly important. However, the stable photoexcitations in HOIP single crystals have not been sufficiently investigated, and it remains unclear whether they correspond to free excitons, bound excitons, free/trapped electron–hole (e–h) pairs, or charged-exciton complexes such as trions^19–22^. The reason for this debate is the relatively small exciton-binding energy in these compounds, which makes it difficult to decide whether or not the excitons dissociate immediately upon light excitation, and then fall into shallow traps known to exist in HOIPs. For proper spectroscopic analysis, it is noteworthy that the exciton species in these compounds have four different quantum substates (namely exciton fine structure, EFS), comprising one ‘dark singlet’ and three ‘bright triplet’ levels that may be split in energy by few 100 μeV^23–28^. Consequently, it is conceivable that quantum beatings (QBs) among the four excitonic states would reveal up to six different oscillation frequencies when applying an external magnetic field, which further splits these states and renders all four states optically active^10,29^. However, only two QB frequencies have been observed^10^, with negligible zero-field frequencies^10^ that imply no zero-field fine-structure splitting, this casts doubt on the exciton involvement in the QB phenomenon. Another issue with the exciton model for explaining the QBs is the photoluminescence (PL) spectrum from HOIPs that rarely shows free exciton emission or emission from e to h pairs associated with the continuum at the band edge. In fact, most of the steady-state PL emission in these compounds originates from sub-bandgap states^30–33^, whose nature has not yet been clearly established.

**Fig. 1:**
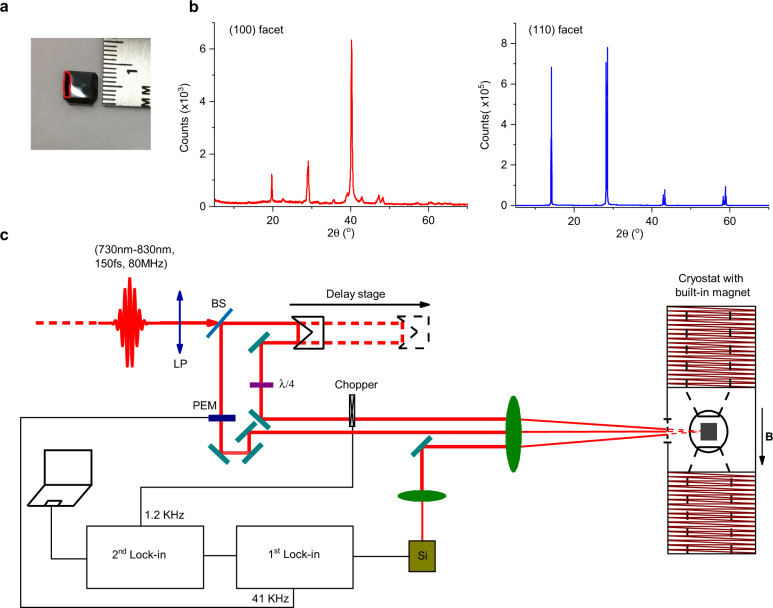
MAPbI_3_ crystal, X-ray diffraction patterns and experimental set-up. **a** MAPbI_3_ single crystal where the (110) surface is in front and (100) surface is on the left (marked by a red line). **b** X-ray diffraction patterns of (100) and (110) crystal facets. **c** Schematic of the experimental apparatus for pump–probe transient photoinduced circular-polarized reflection, c-PPR(t). PEM is a photoelastic modulator for modulating the pump-beam polarization between left and right circular polarizations; λ/4 is a quarter-wavelength plate; LP is a linear polarizer; and BS is a beam-splitter. The MAPbI_3_ crystal was mounted in a cryostat and cooled down to 4 K. An electromagnet generates a magnetic field **B** up to 700 mT parallel to the crystal surface (Voigt geometry).

Here we have used ultrafast spectroscopy at liquid He temperature (4 K) for measuring transient QBs in a high-quality single crystal of the prototypical HOIP, namely the methyl-ammonium lead iodine, MAPbI_3_ (Fig. 1a) in the orthorhombic phase. Specifically, we measured transient spin relaxation and QBs using circularly polarized photoinduced reflectivity (PPR) that are generated due to the Zeeman splitting associated with an applied magnetic field. We found that the spin-relaxation time in the high-quality single-crystalline MAPbI_3_ at 4 K is of the order of ten ns, which enables the observation of several oscillations associated with two different QBs with slow and fast frequencies. Importantly, we found that the QB frequencies at zero field are miniature (i.e., <0.1 GHz), this is in contrast to the exciton explanation for the QB, since the lack of QB at zero field indicates ultra-small EFS that is not the case in HOIPs^24–27^. From this and the different excitation spectra of the slow and fast oscillations, we assign the QBs in MAPbI_3_ to positive and negative trions, for which the Zeeman energy is determined by the lone charge in the trio of particles^34^, as explained in Supplementary Note 3. Consequently from the linear QB-frequency responses with *B*, we obtained the anisotropic g-values of the electron and hole (e&h) in the MAPbI_3_ crystal. The measured anisotropy is in excellent agreement with an 8-band K.P calculation for orthorhombic MAPbI_3_, presented in Supplementary Note 1, and allows us to determine for the key band-structure parameters by fitting to the measured g-factor anisotropy.

## Results

The MAPbI_3_ single crystals in our study were synthesized by a ligand-assisted growth method, which has been demonstrated to tune the facets of the crystals^35^. The grown crystals also have a record-low charge-trap density due to the modulation of ion addition during the solution-growth process. Our experimental set-up for measuring the transient PPR is shown in Fig. 1c. It is a derivative of the well-known degenerate pump/probe technique, where the polarization of the pump beam is modulated by a photoelastic modulator between left (δ^+^) and right (δ^-^) circular polarization (LCP or RCP) for circular PPR (c-PPR). In this scheme the probe beam is circularly polarized (either LCP or RCP) by a quarter-wave plate. The transient change in the probe reflection, ${\triangle R}_{{\delta }^{-}{\delta }^{+}}^{{\delta }^{+}{\delta }^{+}}\,$ = ${R}_{{\delta }^{+}{\delta }^{+}}$ − ${R}_{{\delta }^{-}{\delta }^{+}}$ is recorded (see ‘Methods’ for more details). In contrast to the more traditional pump/probe technique in which the pump intensity is modulated and the measured photoreflectivity (or photoabsorption) is proportional to the photogenerated exciton density, *N*; the c-PPR is proportional to the population difference $({N}_{{\delta }^{+}}-{N}_{{\delta }^{-}})$ between LCP- and RCP-pump excitations.

For our t-PPR measurements, we have used a Ti: sapphire pulse laser having ~150-femtosecond pulse duration at ~80 MHz repetition rate, which could be tuned from 730 nm to 830 nm. The fundamental beam was split into two beams by an 80/20 beam splitter for pump and probe in the degenerate configuration. The pump and probe beams were aligned onto the MAPbI_3_ crystal that was placed inside a cryostat with a built-in electromagnet that delivered a field with strength, *B* up to 700 mT that was applied parallel to the crystal surface (i.e., Voigt geometry), at temperature between 4 K and 300 K. Since the pulsed-laser linewidth (~8 meV) is relatively broad, at resonance condition with the exciton levels in MAPbI_3_, the pump pulse may simultaneously excite spin sublevels of various excitations such as free and trapped excitons and e&h pairs that may lead to transient QBs among their spin sublevels. When measuring QBs at *B* = 0, the transient PPR technique is a unique method for resolving a small ZFS, which may be in the µeV energy range (resulting in few ns QB oscillatory period). At *B* > 2 mT, we found that the QB frequencies change linearly with *B*, from which we obtained the associated g-factors and their anisotropy in the MAPbI_3_ crystal.

**Fig. 2:**
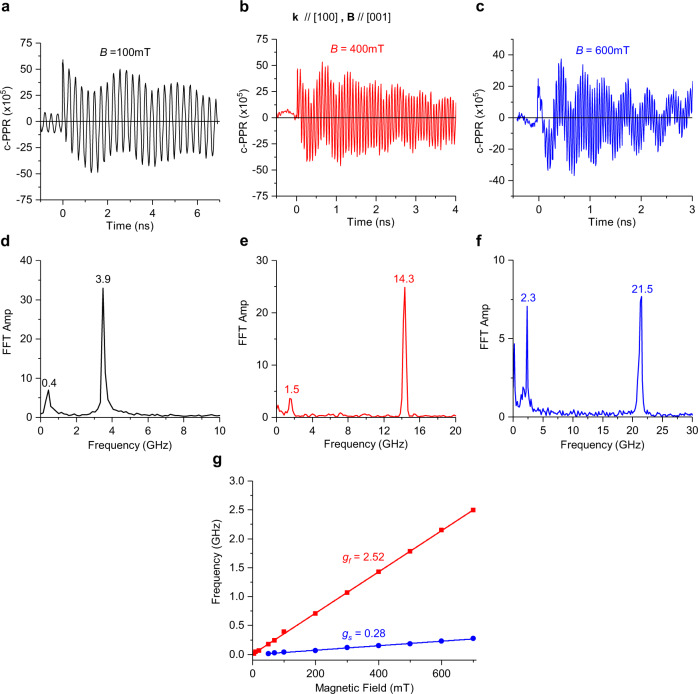
Photoinduced quantum beatings in MAPbI_3_ single crystal at various magnetic field strengths measured at 4 K with light incident along [100] and applied magnetic field along [001]. **a**–**c** Magnetic-field dependence of the c-PPR(t) response measured at 758.5 nm pump/probe beams at various field strengths as denoted, and their corresponding FFT spectra **d**–**f**. **g** The corresponding QB frequencies for the fast (red) and slow (blue) beatings vs. *B* up to 700 mT and the obtained g-values from the various slopes, as indicated.

**Fig. 3:**
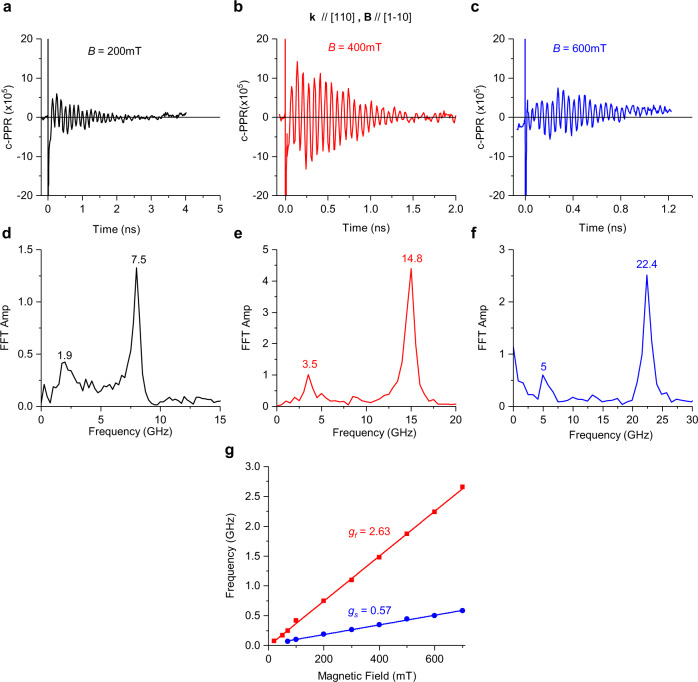
Photoinduced quantum beatings in MAPbI_3_ single crystal at various magnetic-field strengths measured at 4 K with light incident along [110] and applied magnetic field along [1–10]. **a**–**c** Magnetic-field dependence of the c-PPR(t) response measured at 756 nm pump/probe beams at various field strengths as denoted, and their corresponding FFT spectra **d**–**f**. **g** The corresponding QB frequencies for the fast (red) and slow (blue) beatings vs. *B* up to 700 mT and the obtained g-values from the various slopes as indicated.

The transient c-PPR measurements were conducted on a MAPbI_3_ single crystal at cryogenic temperature, where the beams were aligned along two crystal orientations, namely [100] and [110] (Fig. 1b), with an applied magnetic field in the Voigt configuration, in which the field direction is parallel to the crystal surface directed along [001] and $[1\bar{1}0]$, respectively. Figures 2 and 3 show the c-PPR(t) dynamics and their corresponding fast Fourier transform (FFT) spectra measured for different crystal orientations and magnetic-field strengths at ~758 nm (~1.64 eV) pump excitation, which is in resonance with the exciton feature in MAPbI_3_. As seen from the FFT spectra (Fig. 2a–f and Fig. 3d–f), at *B* > 50 mT, we clearly observe two QB frequencies, namely slow and fast, which increase linearly with *B*.

**Fig. 4:**
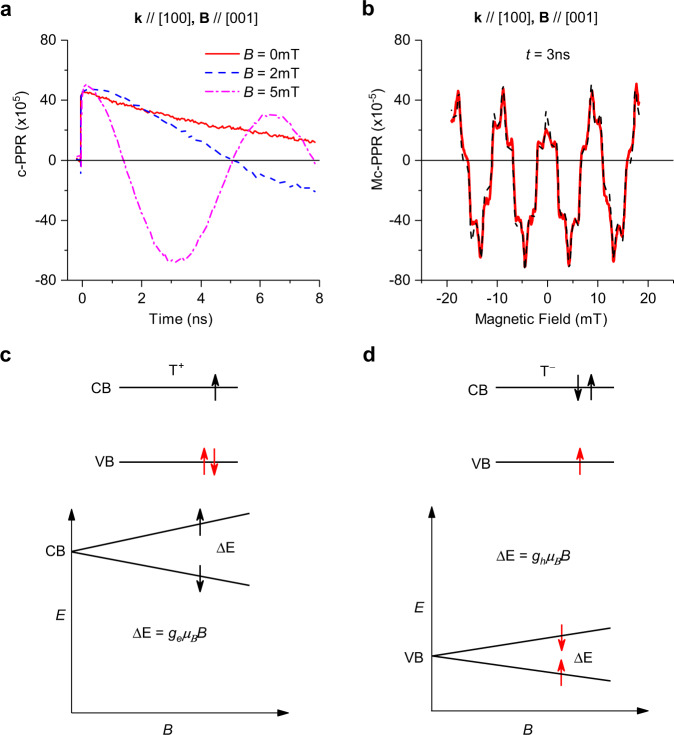
Trion zero-field splitting and Zeeman splitting, and their related quantum beatings at small magnetic field. **a** The c-PPR(t) response in MAPbI_3_ measured on (100) crystal facet at 758 nm pump/probe beams at *B* = 0, 2 mT and 5 mT, respectively, with applied **B** along [001]. **b** The magnetic circular-PPR (Mc-PPR(B)) response measured at fixed delay time, *t* = 3 ns (black dashed line) and its fitting (red line) using Eq. (1). **c** and **d** Schematic representation of positive T^+^ and negative T^−^ trions and their correspondent Zeeman splitting under applied magnetic field. The red (black) arrows represent the holes’ (electrons) spin orientation. Note that the Zeeman splitting is determined by the single particle in the trion since the two spins of the like particles are antiparallel to each other.

In order to study the ZFS of the QB-related photoexcitations, we measured the c-PPR dynamics at zero magnetic field up to 8 ns time delay, as shown in Fig. 4a for measurements on the (100) facet, with magnetic field directed along [001]. It is seen that the c-PPR(t) at *B* = 0 lasts beyond 8 ns with estimated spin lifetime, *τ* > 10 ns. Applying a small *B* = 2 mT causes the c-PPR(t) to cross the zero line at ~5 ns, indicating that there is a very long QB oscillation whose period is comparable to the pulse laser repetition time (*t*_rep_ ~ 12.5 ns here). At increasing *B*, the QB period decreases further (Fig. 4a).

For better studying the ultra-small QB frequency at *B* = 0, we used a different approach, where we take into account the remaining response from the preceding pulses due to the relatively short repetition time, *t*_rep_. In this approach, known in the literature as resonant spin amplification (RSA)^36^ instead of scanning the delay time, *t*, in measuring the c-PPR(t) response, we scanned the *B* field at a constant delay time, *t* and measured the magnetic PPR response; we name this c-PPR version ‘magnetic field circular PPR’, or Mc-PPR(B,t), as seen in Fig. 4b. The observed oscillations in the Mc-PPR(B) can be analyzed by the equation^36^

1 $${{{{{\rm{Mc}}}}}}-{{{{{\rm{PPR}}}}}}\left(B,t\right)=	\, \mathop{\sum}\limits_{n}\Theta (t+n{t}_{{{{{\rm{rep}}}}}})\mathop{\sum}\limits_{i=f,s}{A}_{i}{{{{{{\rm{e}}}}}}}^{-{\Gamma}_{i}\left(t+n{t}_{{{{{\rm{rep}}}}}}\right)}\\ 	 \cos\left[\frac{({g}_{i}{\mu}_{B}B+\Delta{{{{{\rm{E}}}}}})}{{{\hslash}}}(t+n{t}_{{{{{\rm{rep}}}}}})\right]$$

where the index *n* runs over the present and preceding laser-pump pulses that contribute to the Mc-PPR response. The index *i* represents the fast and slow contributions for which ${\Gamma }_{f,s}$ and *g*_*f,s*_ are their spin-relaxation rate and Landé *g*-factors, respectively. The important parameter in Eq. (1) that we measure is ΔE, which is the ZFS energy of the related photoexcitations. The obtained Mc-PPR(B) responses were fitted by adjusting the parameters *A*_*i*_ and ΔE, while fixing *g*_*f/s*_ values as obtained from the c-PPR(t) dynamics at various *B* fields (Fig. 2), as well as the *B*-dependent spin-relaxation rate Γ_*i*_(*B*), which were measured separately (see below). A good fit to the experimental Mc-PPR(B) response was obtained using ΔE_(100)_ = 40 neV (nano eV). This value is in good agreement with the ultra-small ZFS energy that is obtained from the extrapolation to *B* = 0 of the QB frequencies vs. *B* in the c-PPR(*t*) (Fig. 2g).

The fact that the ZFS is negligibly small indicates that excitons are not the photoexcitations underlying the observed QBs^37^. In Supplementary Note 2, we show the calculated magnetoexciton fine structure for MAPbI_3_ and the resulting simulated c-PPR signatures; the participation of excitons in the QB response is shown in the S.I. to lead to two distinct beat frequencies at zero-applied magnetic field with two additional beat frequencies emerging with increased magnetic field due to magnetic activation of the dark exciton. This behavior stands in stark contrast to the experimental observations here. We thus conclude that the QBs in MAPbI_3_ are due either to electrons and holes or to positive and negative trions, where the excitons are trapped by photogenerated holes and electrons, respectively, or to separately localized resident carriers remaining after trion recombination^38–40^.

MAPbI_3_ is known to have photogenerated charges with long lifetime even at room temperature, let alone at 4 K. Therefore, there is a large background density of photogenerated electrons and holes that are due to the pump excitation that can trap the photogenerated excitons. Note that trions comprise a bound state of three particles. A negative trion, T^−^ is composed of one hole and two electrons (hee), whereas a positive trion, T^+^ is composed of one electron and two holes (ehh), as schematically shown in Fig. 4c and d, respectively. It is known that the overall exchange interaction of trions among the three bound particles, which determines the trion ZFS energy, in fact vanishes in the trion ground state. This was shown in ref. ^41^ and discussed in Supplementary Note 3. Moreover, when trions are photogenerated, the spins of the two like-charge particles need to align antiparallel to each other (see Supplementary Fig. 5 and related detailed discussion in Supplementary Note 3). Therefore, the Zeeman splitting of a trion is determined by the lone charge in the particles’ trio^34^. In particular, the Zeeman splitting of T^+^ is governed by the electron g-factor, ${g}_{e}$, since ${H}_{Z}={g}_{e}{\mu }_{B}B$; whereas the Zeeman splitting of T^−^ is governed by the hole g-factor, ${g}_{h}$. Consequently, in either case (trions or residual carriers left behind after trion decay), the g-factors extracted from the QB frequencies vs. *B* are those of the electron and hole. It is not possible to assign the particular g-value to either particle, but when comparing with calculations^10, 29^, it is likely that the larger g-value (fast oscillation) is due to the electron in the T^+^; whereas, the smaller g-value (slower oscillation) is due to the hole in T^−^. From the QBs measured along different crystal orientations, we thus obtain the anisotropic g-factors in MAPbI_3_ to be $\big|{g}_{[001]}^{e}\big|$  = 2.52 and $\big|{g}_{[1\bar{1}0]}^{e}\big|$ = 2.63 for the electron; whereas $\big|{g}_{[001]}^{h}\big|$ = 0.28 and $\big|{g}_{[1\bar{1}0]}^{h}\big|$ = 0.57 for the hole; where the subscripts refer to the direction of the applied magnetic field. The relation $\big|{g}_{e}\big| \; > \; \big|{g}_{h}\big|$ observed here is consistent with the K.P model calculation of ref. ^29^, which was performed for tetragonal MAPbI_3_. However, our experiments are performed at liquid helium temperature at which MAPbI_3_ is known to be in the orthorhombic phase, so that the model of ref. ^29^ is not quantitatively applicable.

**Fig. 5:**
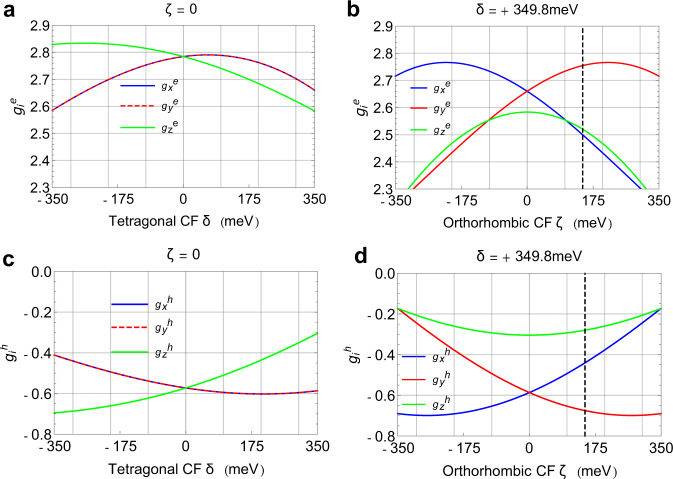
Calculated electron and hole g-factors in MAPbI_3_. **a** and **c** The calculated electron and hole g-factors in tetragonal MAPbI_3_ as a function of the tetragonal crystal field (CF), $\delta$, with orthorhombic CF $\zeta =0$. **b** and **d** The electron and hole g-factors in orthorhombic MAPbI_3_ for fixed tetragonal CF $\delta$=+349.8 meV, determined by fit to the measured g-factors, as a function of the orthorhombic CF, $\zeta$. The best-fit value for $\zeta$ is +147.7 meV, marked in panels (**b**, **d**) with a vertical dashed black line. The subscripts *x*, *y* and *z* on the g-factor components denote the [1,0,0], [0,1,0] and [0,0,1] crystallographic directions. In the calculations shown, the bandgap ${E}_{g}$, the SOC split-off parameter $\Delta$, the Kane energy ${E}_{p}$, and Luttinger’s magnetic parameter $\kappa$ have the values shown in Table 1. From the values plotted, the g-factors along the $[1\bar{1}0]$ direction are obtained as^29^ ${g}_{[1\bar{1}0]}^{e}=\scriptstyle{\sqrt{1/2{\left({g}_{{{{{{\rm{x}}}}}}}^{e}\right)}^{2}+1/2{\left({g}_{{{{{{\rm{y}}}}}}}^{e}\right)}^{2}}}$; ${g}_{[1\bar{1}0]}^{h}=-\scriptstyle{\sqrt{1/2{\left({g}_{x}^{h}\right)}^{2}+1/2{\left({g}_{y}^{h}\right)}^{2}}}$.

**Fig. 6:**
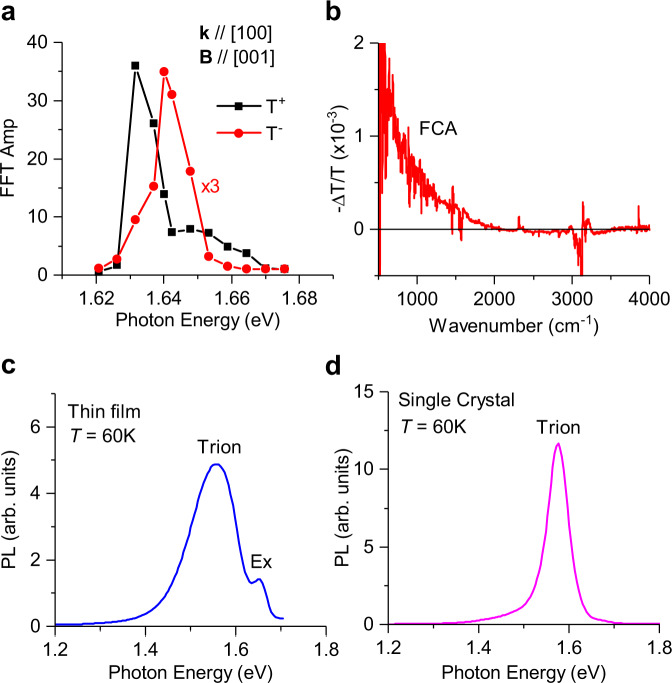
The quantum-beating excitation spectra, photoinduced free carrier-absorption spectrum, and photoluminescence spectra. **a** The quantum-beating FFT-amplitude spectra of the fast (T^+^) and slow (T^−^) oscillations, referred to as QB-excitation spectra, measured at *B* = 400 mT on (100) facet with applied **B** field along [001]. The QB-excitation spectra peak at 1.63 eV for the fast oscillations and 1.64 eV for the slow oscillations, both energies are below the exciton optical transition at 1.65 eV. **b** The photoinduced absorption (PA) spectrum of MAPbI_3_ thin film measured at 60 K using a FTIR spectrometer, excited at 470 nm, which shows free carrier-absorption (FCA) spectrum. **c** The PL-emission spectrum of a MAPbI_3_ thin film measured at 60 K that shows two bands: the higher-energy band ~1.65 eV is due to free exciton emission and the lower, broader band originates from trapped excitons in charged shallow traps (or trions). **d** The PL spectrum of a MAPbI_3_ single crystal measured at 60 K, which is predominantly due to trions.

We therefore developed an 8-band K.P model for orthorhombic HOIPs such as MAPbI_3_. This model is outlined in the Methods section and described in detail in Supplementary Note S1. In short: Employing the quasi-cubic approach of ref. ^27^, we determine the band-edge Bloch functions of the conduction band by diagonalization of the K.P Hamiltonian, that also includes the spin-orbit interaction and the crystal field contributions^27,29^ which break the symmetry of the [100], [010] and [001] directions in the orthorhombic structure. Writing the full 8-band K.P Hamiltonian that includes k-dependent terms, see Supplementary Eq. S17–S18 in SI, and adding the magnetic Hamiltonian^42^ given in Supplementary Eq. S20 as a perturbation (see Methods), we evaluate the effective Zeeman Hamiltonian for the lowest conduction band and the valence band using Löwdin’s partition method^43^ and thereby determine the electron and hole g-factors. While the g-factors are isotropic in the cubic-phase HOIPs, in tetragonal or orthorhombic perovskites, the symmetry between the orthogonal [100], [010] and [001] directions is broken, leading to anisotropic g-factor tensor components as shown in Fig. 5. Note that the six principal g-factor components (x, y and z values for the electron and the hole, respectively, where x, y and z align to [100], [010] and [001]) are fully determined by six parameters: the bandgap, ${E}_{g}$; the Kane energy^27^, ${E}_{p}$ the spin–orbit split-off parameter, $\Delta$; the tetragonal^29^ and orthorhombic^27^ crystal field parameters $\delta ,\zeta$, respectively, and Luttinger’s magnetic parameter^42^, $\kappa$. The bandgap is determined from the PL spectra (see Fig. 6), while the split-off parameter is known from independent measurements and calculations^29^, enabling determination of the remaining four parameters ${E}_{p}$, $\delta ,\zeta$ and $\kappa$ by measuring the electron and hole g-factors along two axes of the crystal as described above. In Table 1, we show the values of these parameters calculated by best fit to the measured g-factors; the resulting calculated g-factors match the experimental values.

**Table 1:** Band-structure parameters for orthorhombic MAPbI_3_.

Parameter	Value	Source
Kane energy ${E}_{p}$	13.884 eV	This work
Tetragonal CF $\delta$	349.8 meV	This work
Orthorhombic CF $\zeta$	147.7 meV	This work
Luttinger’s magnetic parameter $\kappa$	0.206	This work

Using the expressions in Supplementary Table 1 of Supplementary Note 1, the Kane energy, ${E}_{p}$, the crystal field parameters $\delta$ and $\zeta$ and Luttinger’s magnetic parameter $\kappa$ are uniquely determined by measurement of the electron and hole g-factors along two directions. The measured values are taken as ${g}_{Z}^{h}=-|{g}_{[001]}^{h}|$ =  *−*0.28; ${g}_{[1\bar{1}0]}^{h}=-0.57$ for the holes, ${g}_{Z}^{e}={g}_{[001]}^{e}=2.52$ and ${g}_{[1\bar{1}0]}^{e}=2.63$. For these calculations, the bandgap, ${E}_{g}$, is taken as 1.65 eV corresponding to the measured exciton energy and the SOC parameter $\Delta =1.4$ eV from ref. ^24^. The calculated g-factor values match the experimental values.

The resulting values for the Kane energy and Luttinger’s magnetic parameter are close to the corresponding values for tetragonal MAPbI_3_^29^ as expected in a quasi-cubic system. From the fit, we find that the tetragonal crystal field is positive, in agreement with density-functional theory (DFT) calculations of band structure of tetragonal MAPbI_3_ reported in refs. ^44,45^, the hybrid DFT calculations for both tetragonal and orthorhombic MAPbI_3_ reported in ref. ^27^ and the 16-band K.P model for tetragonal MAPbI_3_ reported in ref. ^24^, but contrary to the analysis in ref. ^29^ (see the illuminating discussion in ref. ^26^). The magnitude, *δ* = + 349.8 meV, is in line with the range of the values calculated using DFT or hybrid DFT ~ + 100–240 meV^24,27^. The orthorhombic crystal field is found to be $\zeta$ = +147.7 meV, which agrees in sign but is larger in magnitude than the value (+82 meV) calculated using hybrid DFT in ref. ^27^. The band-edge energies and the exciton fine structure that result from these parameters are shown in Supplementary Figs. 1, 2.

In order to test the trion scenario, we measured the excitation dependence of the two QBs. We changed the pump–probe wavelength and measured the QBs at fixed *B* = 400 mT on (100) crystal facet with **B**-field direction along [001]. Subsequently, we performed a Fast Fourier transform of the periodic c-PPR(t) response to obtain the FFT component of the slow and fast QB oscillations. The excitation dependences of the QB FFT components are shown in Fig. 6a. It is seen that the excitation spectrum of the slow (hole) and fast (electron) is different. In particular, they peak at different energies; the fast QB component peaks at ~1.63 eV, whereas the slow QB component peaks at ~1.64 eV. Both energies are below the MAPbI_3_ exciton energy, *E*_*x*_ at this temperature (*E*_*x*_ ~ 1.65 eV) as deduced from the free exciton PL band (Fig. 6c)^32^. From the different excitation spectra for the slow and fast oscillations we conclude that the QBs cannot be due to free e–h pairs, which should show overlapping excitation spectra; and this supports the trion interpretation. Consequently, we calibrate the positive and negative trion optical transition in MAPbI_3_ crystal to be at 1.63 eV and 1.64 eV, respectively.

In further support for this assignment we note that the sample is constantly illuminated by the pulsed excitation at low temperature, where the resulting photoexcitations do not decay in-between adjacent pump pulses. Under these conditions steady state photocarrier density may be formed in the illuminated area of the crystal that subsequently leads to trion photogeneration, especially at the resonance excitation condition. To check this assumption, we performed steady state photoinduced absorption (PA) measurement in MAPbI_3_ film using a cw-laser illumination and probed by a FTIR spectrometer (see Methods). In this experiment, the laser light was slowly modulated and the change, ΔT in the transmission spectrum, *T* was monitored, where PA = −ΔT/*T*. Figure 6b shows the PA spectrum of MAPbI_3_ measured at low temperature. It clearly shows the PA spectrum of free carriers, namely free carrier absorption (FCA) that increases towards small photon energy, *ħω*, as *ω*^−2^. In the PA experiment it can be easily shown that −ΔT/*T* = Δα*d*; where *d* is the film’s thickness, Δα is the induced absorption that is given by the relation Δα = *Nσ*, where *N* is the photocarriers density and *σ* is the optical cross section. Since we know the film thickness *d* = 100 nm, we estimate Δα from the PA band to be ~100 cm^−1^ at *ħω* = 1000 cm^−1^. Consequently, from the previous FCA cross-section value at 1000 cm^−1^, *σ* ~ 10^−16^ cm^2^, we estimate the steady-state photocarrier density in MAPbI_3_ at liquid He temperature to be *N* ~ 2 × 10^17^ cm^−3^. Taking into account that the average power in the pulsed experiment is about an order of magnitude larger than that used in the cw measurements, we estimate a background steady state photocarriers density of ~10^18^/cm^3^, which is much larger than the carrier density in equilibrium at 4 K, or the density of impurities. This high carrier density is sufficiently large to capture most photoexcited excitons in the sample^46^. As a result, the photoluminescence spectrum in the MAPbI_3_ crystal at low temperatures is dominated by trion emission, as seen in Fig. 6d and Supplementary Fig. 7, which is further red-shifted possibly due to the photon-recycling process^47^. The PL emission of the MAPbI_3_ crystal at 10 K as a function of the laser excitation intensity (*I*_*L*_) shows a dominant trion band that grows as (*I*_*L*_)^1.5^ as shown in Supplementary Fig. 7b. In addition, the PL emission band blue-shifts with increasing temperature and changes abruptly at the tetragonal-to-orthorhombic phase-transition temperature^32^. Taken together, these measurements support our assignment of photogenerated trions in MAPbI_3_.

To provide additional experimental evidence for trions, we measured the QBs of a pristine MAPbI_3_ film and compared these to those of a seemingly n-type doped film; the doping was achieved by soaking the MAPbI_3_ film in benzyl viologen (BV) solution in toluene for ~one minute, where BV is an electron-donating molecule (see ref. ^48^). It is known that MAPbI_3_ can self-dope according to the precursor ratio of MAI to PbI_2_ (see ref. ^49^). Our ‘as grown’ MAPbI_3_ films fall in the category of p-doped, so that the light doping achieved by soaking in BV actually compensates the p-type dopants that are originally in the film. As shown in Supplementary Fig. 8, both QB amplitudes increase upon compensation, because the steady-state photocarrier density background increases in ‘intrinsic’ semiconductors. The mere fact that the QB amplitude changes upon ‘doping’ supports our assignment for the trion quantum beatings, otherwise, there should not be any dependence of the QB amplitude on the Fermi level in the film.

**Fig. 7:**
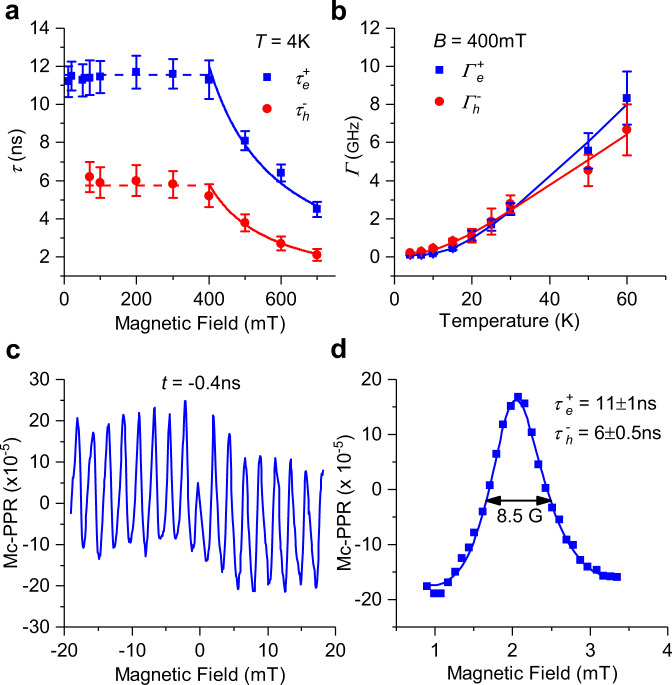
Magnetic field and temperature dependencies of the spin dynamics in MAPbI_3_ single crystal measured on (100) facet with B // [001]. **a** Magnetic-field dependence of the spin-decay time, *τ*(*B*) for the T^+^ and T^−^ species (${{\tau }}_{e,h}^{+,-}$) up to 700 mT measured at 4 K. Error bars derived from the least mean-square fit of the c-PPR dynamics in Fig. 2 and Supplementary Fig. 9 using Eq. (2). **b** Temperature dependence of the spin-decay rate ${\Gamma }_{e,\left(h\right)}^{+,\left(-\right)}(T)$ for the T^+^ and T^−^ species measured at *B* = 400 mT. Error bars derived from the least mean-square fit of the c-PPR dynamics in Supplementary Fig. 12 using Eq. (2). **c** The B-scan of the QB signal (Mc-PPR(*t*)) measured at a fixed delay time *t* = −0.4 ns, at 756 nm, in the B interval from −20 mT to 20 mT. This QB technique is known in the literature as ‘resonance spin amplification’ (RSA) ref. ^36^, which has been used to obtain the spin-relaxation time, *τ* (or ${\tau }_{2}^{* }$) in materials in which *τ* > *t*_rep_, the pulse-to-pulse time interval. **d** The RSA(B) resonance around B = 2 mT (blue squares); the line through the data points is a fit using Eq. (1) in the text, from which we obtained the spin-relaxation times of the T^+^ and T^−^ (${{\tau }}_{e,h}^{+,-}$) as shown. The resonance FWHM is denoted.

We further use the QB spectroscopy to investigate the spin dynamics of the trions in MAPbI_3_. First, we fit the two QB oscillations using the equation

2 $${A}_{1}{e}^{\frac{-t}{{\tau }_{1}}}{{\cos }}({2{\pi }f}_{1}t+{{\phi }}_{1})+{{{{{{\rm{A}}}}}}}_{2}{e}^{\frac{-t}{{\tau }_{2}}}{{\cos }}({2{\pi }f}_{2}{{{{{\rm{t}}}}}}+{{\phi }}_{2})$$

where *f*_1_ and *f*_2_ are the two QB frequencies (namely T^+^ and T^−^) that were extracted from the FFT of the transient dynamics, and *τ*_1_ and *τ*_2_ are the QB-decay lifetimes. This was done at different magnetic fields at 4 K (Fig. 7a and Supplementary Figs. 9–11) and at various temperatures at a fixed field of 400 mT (Fig. 7b and Supplementary Fig. 12). As depicted in Fig. 7a, the T^+^ and T^−^ spin-relaxation times, ${\tau }_{e}^{+}$ and ${\tau }_{h}^{-}$ measured along [100] crystal orientation, are nearly constant at field *B* < 400 mT, but steeply decrease as 1/*B* at *B* ≧ 400 mT. We note that the ${\tau }_{e}^{+}$ saturation at small magnetic fields could be due to the limit of our pulse-repetition time (*t*_rep_ ~ 12.5 ns). Therefore, for obtaining the correct T^+^ spin lifetime, we measured the Mc-PPR(B,t) response (i.e., the RSA method discussed above) at negatively fixed delay time *t* = −400 picoseconds (ps) (see Fig. 7c). This RSA method has been used before to extract ${\tau }_{2}^{* }$ for electrons in GaAs (${\tau }_{2}^{* }$ ≈ 130 ns, ref. ^36^) and CdTe quantum wells (${\tau }_{2}^{*}$≈ 30 ns, ref. ^50^) even though that the pulse-to-pulse time period, *t*_rep_ was much smaller than the extracted ${\tau }_{2}^{* }$ in both cases. Given that *t*_rep_ is actively stabilized to 1.5 ps, and the magnetic field is measured to 0.1 Gauss, the RSA method is able to determine the spin lifetime as long as 5 μs, even that *t*_rep_ is ~2 orders of magnitude shorter (see ref. ^36^. and ref. ^40^).

As seen in Fig. 7c, the RSA response at *t* = −400 ps is comprised of a sequence of RSA resonances having full width at half maximum (FWHM) as narrow as ~1 mT. We note that ${\tau }_{2}^{* }$ may be directly obtained from the width of the RSA resonances, where longer ${\tau }_{2}^{* }$ corresponds to narrower width. For example, for electrons in GaAs with |${g}_{e}\,$| = 0.44, the FWHM of ~0.3 mT corresponds to ${\tau }_{2}^{* }$ of 130 ns. Similarly, in CdTe QWs with |${g}_{e}\,$| =1.64, the FWHM of ~0.6 mT results in ${\tau }_{2}^{* }$ ~ 30 ns. In the MAPbI_3_ crystal with |${g}_{[001]}^{e}\,$| = 2.52, the FWHM at *B* = 2 mT is ~ 1mT, which gives ${\tau }_{2}^{* }$ of ~10–15 ns. In order to get a precise ${\tau }_{2}^{* }$ for T^+^ (${\tau }_{2}^{* }={\tau }_{e}^{+}$) we fitted the RSA response at *B* = 2 mT using Eq. (1) and obtain ${\tau }_{e}^{+}$ = 11 ± 1 ns, which is consistent with the ${\tau }_{e}^{+}$ value extracted from the time-scan experiments. In addition, we also explored the RSA resonances at larger *B*. We observed that the FWHM of the RSA resonances at *B* < 400 mT is the same as for *B* = 2mT, confirming that ${\tau }_{e}^{+}$ saturation at small fields extracted from the time-scan experiment is correct.

The steep decrease of τ(*B*) was previously observed in CdTe quantum wells^40,50^ and attributed to an inhomogeneous dephasing of an ensemble of carrier spins caused by a variation of Larmor precession frequencies due to dispersion of the carriers’ *g*-factor, Δ*g*. This can be written as

3 $$\frac{1}{{\tau }_{2}^{* }}=\frac{1}{{\tau }_{0}}+\Theta \left({B}_{0}\right)\frac{\triangle {{{{{\rm{g}}}}}}{\mu }_{B}B}{{{\hslash }}},$$

Here ${\tau }_{2}^{* }$ = ${\tau }_{e}^{+}$ or ${\tau }_{h}^{-}$, ${\tau }_{0}$ is the spin lifetime at zero and small B fields, and $\Theta \left({B}_{0}\right)$is a step function that describes the saturation of ${\tau }_{2}^{* }$ for *B*_*0*_ < 400 mT. From the 1/*B* fitting of experimental data for *B* ≥ 400 mT (Fig. 7a), we obtain $\triangle {{{{{{\rm{g}}}}}}}_{{{{{{\rm{e}}}}}}}^{+}$ = 0.005 and $\triangle {{{{{{\rm{g}}}}}}}_{{{{{{\rm{h}}}}}}}^{-}$ = 0.011 for **B** along [001] crystal orientation. We note that the observed saturation of ${\tau }_{e}^{+} \sim$ 11 ns and ${\tau }_{h}^{-}$ ~ 6 ns sets a lower limit of ${\tau }_{0}$ for T^+^ and accordingly for T^−^.

Furthermore, we found that the spin-relaxation rates for both T^+^ and T^−^ increase with the temperature *T* up to 60 K (Fig. 7b and Supplementary Fig. 12). For *T* > 60 K, we do not observe any QB signals, which could be due to fast disintegration of trions at higher temperatures. In this case, we estimate the trion-binding energy to be of the order of 5 meV. The *τ*(*T*) response supports the Elliot–Yafet (EY) spin-relaxation mechanism, which arises from spin–orbit-related scattering collisions with phonons^51^. In this case, the spin-relaxation rate is proportional to the phonon-scattering rate Γ_*p*_. Since the occupancy, 〈*n*〉, of optical phonon increases with *T*, the carrier–phonon-scattering rate increases, leading to an increase in the spin-relaxation rate Γ (=1/*τ*). We fitted the temperature-dependent rates using the function ${\Gamma }^{+,(-)}$ = ${\Gamma }_{0}^{+,(-)}+{\Gamma }_{\omega }^{+,(-)}\frac{1}{{e}^{\frac{\hslash \omega 0}{{K}_{B}T}}-1}$, where ${\Gamma }_{0}^{+,(-)}$ is the temperature-independent scattering rate from defects and impurities, ${\Gamma }_{\omega }^{+,(-)}$ is the scattering rate from phonons and *ω*_*0*_ is a typical optical phonon frequency. Good fits for ${\Gamma }_{e}^{+}$(*T*) and ${\Gamma }_{h}^{-}$(*T*) have been obtained with ${\Gamma }_{0}^{+(-)}$ = 0.09 (0.2) ± 0.005 (0.02) (GHz), ${\Gamma }_{{\omega }}^{+(-)}$ = 13 (10) ± 2 (1) (GHz) and ${\hslash \omega }_{0}$ = 5 ± 3 meV (see Fig. 7b). That Γ_0_ ≪ Γ_*ω*_ indicates that carrier scattering with defects/impurities is negligibly small in this high-quality MAPbI_3_ single crystal. The fitting also shows that the optical phonon modes having energy <10 meV are the dominant scatterers that influence the trion spin-relaxation kinetics.

In conclusion, the quantum beatings observed in a high-quality MAPbI_3_ single crystal at cryogenic temperature originate from positive and negative trions for which the beating frequencies increase linearly with the applied magnetic field. This may be interpreted as the Larmor precession frequencies of electron (hole) in the positive T^+^ (negative T^−^) trions or to the residual carriers left after trion decay. The obtained Landé *g*-factors of both electrons and holes show a significant anisotropy when the magnetic field was directed along two different crystal axes [001] and [$1\bar{1}0$], respectively. In addition, the unexpected long spin-relaxation time τ of T^+^ trions ~11 ns at liquid He temperature could make the MAPbI_3_ single crystal a potential candidate for quantum information science.

## Methods

### Sample preparation

In our studies, we have used both single crystals and thin films of MAPbI_3_. The single crystals were grown by typical ITC method^35^ in which we mixed CH_3_NH_3_I and PbI_2_ in a 1:1 molar ratio in gamma-butyrolactone (GBA) to form the precursor solution with a concentration of 1.23 mol/ml. The precursor solution was stirred overnight at 80 °C. Subsequently, the solution was filtered using PTFE filter with 0.22-μm pore size. Finally, the precursor solution was kept in a large beaker, which was half-filled with mineral oil and placed on a hot plate at 95 °C for about 2 days. Small MAPbI_3_ particulates were harvested as seed crystals. By placing a seed crystal in 10 mL of the same precursor solution and keeping it at 95 °C for another 2 days, the seed grew into a large MAPbI_3_ crystal of mm size. For larger crystals, the latter step was repeated with the large crystals used as the new seeds.

Thin films were prepared using the standard procedure in which a precursor solution of CH_3_NH_3_I_3_ and PbCl_2_ in a molar ratio of 3:1 in N,N-dimethylformamide was used to form a concentration of 0.8 mol/ml. The precursor solution was stirred on the hotplate at 50 °C overnight. The solution was cooled to room temperature and spin-coated on glass substrates at 3000 rpm for 60 sec. The resulting film was then annealed at 100 °C for 2 h.

### Characterization of the crystal structure

XRD measurements of single-crystal MAPbI_3_ (Fig. 1) were performed using a Bruker D2 Phaser X-ray diffractometer in 2-theta configuration (see S.I.). The flat face of the MAPbI_3_ crystal was placed facing down on a flat surface of the sample-holder ring, while the remainder of the ring was covered with amorphous clay to form a mold around the crystal, such that only the intended face of the crystal remained uncovered. The X-ray source was the K-$\alpha$ emission from a Cu target. A divergence slit of 0.2 mm was placed in the input-beam path and an anti-scatter screen above the sample provided efficient measurements. The measurements were done with step resolution of 0.022˚ over the continuous 2-theta range of 10˚–70˚. The obtained spectrum was compared with that in the literature to determine the crystalline orientation of the measured face. Similar procedure was repeated along other flat faces of the crystal, and the corresponding orientation axis was determined.

### Transient circular-PPR spectroscopy

The transient circular-PPR technique is a derivative of well-known optical pump/probe correlation spectroscopy in which only the polarization of the pump beam is modulated with a PEM (i.e., photoelastic modulator) at 41 kHz between left and right circular polarizations. The probe beam was circularly (linearly) polarized for circular (linear) PPR. The pump and probe beams were split from the output of a Ti: sapphire laser (Spectra-Physics) with pulse duration of 150 fs and 80 MHz repetition rate that could be continuously tuned from 730 nm to 810 nm. In order to minimize the large scattering intensity from the stronger pump beam, we used a double lock-in technique in which the probe beam was also modulated by a mechanical chopper at 1.2 kHz. The pump and probe beams having average intensity of ~20 Wcm^−2^ and ~3 Wcm^−2^, respectively, were aligned through various optical components to spatially and temporally overlap onto a small area of the samples with spot size of ~100 µm. The probe-beam path length was extended by a mechanically delayed stage up to ~8 ns. For the probe-beam intensity detection, we used a silicon photodiode connected to the first lock-in amplifier that was externally synchronized with the PEM at 41 kHz. The second lock-in amplifier was externally synchronized with the chopper frequency at 1.2 kHz. The crystal was placed in a cryostat that controlled the temperature between 4K and 300K. A magnetic field, **B** from an electromagnet with strength, *B* up to 700 mT, was applied in the direction parallel to the crystal chosen surface (i.e., Voight configuration).

### Photoinduced-absorption spectroscopy

Photoinduced absorption (PA) in the mid-IR spectral range was measured using a Fourier-transform infrared spectrometer (FTIR, Thermo Scientific) having an external detecting system as a probe in the spectral range of 500–4000 cm^−1^. The film was excited at *ħω* ≈ 2.77eV using a diode laser of which beam was modulated at 50 mHz using a shutter controlled by a function generator. The change, ΔT in the IR transmission spectrum, *T* induced by the pump-beam excitation was signal-averaged over 5000 scans. The PA spectrum was subsequently calculated as −ΔT/*T*.

### Calculation of the g-factors in orthorhombic MAPbI_3_

To describe the anisotropic electron and hole g-factors in orthorhombic MAPbI_3_, we employ an 8-band K.P model based on the quasi-cubic approach of ref. ^27^. The band-edge valence-band function Bloch functions are represented as the 2-fold degenerate states with S-orbital symmetry, while for the conduction bands, the Bloch functions have orbital P-symmetry^27,44^. To find the band-edge Bloch functions, we diagonalize the conduction-band Hamiltonian, including the spin–orbit interaction, ${H}_{{LS}}=\frac{2}{3}\Delta {{{{{\bf{L}}}}}}{{{{{\boldsymbol{\cdot }}}}}}{{{{{\bf{S}}}}}}$, whose strength is given by $\Delta$, the spin–orbit split-off parameter that separates the upper-$J=3/2$ derived conduction bands from the lower $J=$ ½-derived conduction bands in the cubic crystal structure. We include as well the crystal field Hamiltonian^27,29^, ${H}_{{CF}}$, which breaks the symmetry of the **x**,**y**,**z** directions in the orthorhombic structure. This is given by^27^

4 $${H}_{{CF}}=\left(\zeta -\frac{\delta }{3}\right){L}_{x}^{2}+\left(-\zeta -\frac{\delta }{3}\right){L}_{y}^{2}+\frac{2}{3}\delta {L}_{z}^{2},$$

where ${L}_{x},{L}_{y},{L}_{z}$ are the *x, y, z* components of the orbital angular momentum operator, and the tetragonal^29^ and orthorhombic^27^ crystal-field parameters $\delta$, $\zeta$ reflect symmetry breaking relative to the cubic phase in the ${{{{{\bf{z}}}}}}$ and in the ${{{{{\bf{x}}}}}},{{{{{\bf{y}}}}}}$ directions, respectively. Diagonalization of ${H}_{{LS}}+{H}_{{CF}}$ results in Bloch eigenfunctions for the lower conduction bands that can be represented in the general form^27^

$${u}_{1}^{{{{{{\mathcal{C}}}}}}}=-{{{{{{\mathcal{C}}}}}}}_{Z}Z\uparrow -\left({{{{{{\mathcal{C}}}}}}}_{X}X+i{{{{{{\mathcal{C}}}}}}}_{Y}Y\right)\downarrow ,$$

5 $${u}_{2}^{{{{{{\mathcal{C}}}}}}}=-\left({{{{{{\mathcal{C}}}}}}}_{X}X-i{{{{{{\mathcal{C}}}}}}}_{Y}Y\right)\uparrow +\,{{{{{{\mathcal{C}}}}}}}_{Z}Z\downarrow ,$$

where the symbols $X,Y,Z$ denote orbital functions that transform like *x, y, z* under rotations, while ${{{{{{\mathcal{C}}}}}}}_{X},{{{{{{\mathcal{C}}}}}}}_{Y},{{{{{{\mathcal{C}}}}}}}_{{{{{{\rm{Z}}}}}}}$ are real-valued c-numbers determined by numerical diagonalization and subsequent Gram–Schmidt orthogonalization; these coefficients are functions of the crystal-field parameters $\delta ,\zeta$ as well as the spin–orbit split-off parameter, $\Delta$. Similarly, the energies of the upper heavy- and light-electron-band edges are found by numerical diagonalization of ${H}_{{LS}}+{H}_{{CF}}$. The corresponding Bloch functions can be shown to have the following general forms: for the heavy-electron^52^ band,

$${u}_{1}^{{{{{{\mathcal{H}}}}}}}=-\left({{{{{{\mathcal{H}}}}}}}_{X}{{{{{\rm{X}}}}}}+{{{{{\rm{i}}}}}}{{{{{{\mathcal{H}}}}}}}_{Y}Y\right)\uparrow +{{{{{{\mathcal{H}}}}}}}_{Z}Z\downarrow ,$$

6 $${u}_{2}^{{{{{{\mathcal{H}}}}}}}={{{{{{\mathcal{H}}}}}}}_{Z}Z\uparrow +\left({{{{{{\mathcal{H}}}}}}}_{X}X-{{{{{\rm{i}}}}}}{{{{{{\mathcal{H}}}}}}}_{Y}Y\right)\downarrow ,$$

where the real-valued coefficients ${{{{{{\mathcal{H}}}}}}}_{X},{{{{{{\mathcal{H}}}}}}}_{Y},{{{{{{\mathcal{H}}}}}}}_{{{{{{\rm{Z}}}}}}}$ are found numerically; and for the light-electron^52^ band,

$${u}_{1}^{{{{{{\mathcal{L}}}}}}}={{{{{{\mathcal{L}}}}}}}_{Z}Z\uparrow -\left({{{{{{\mathcal{L}}}}}}}_{X}X+{{{{{\rm{i}}}}}}{{{{{{\mathcal{L}}}}}}}_{Y}Y\right)\downarrow ,$$

7 $${u}_{2}^{{{{{{\mathcal{L}}}}}}}=\left({{{{{{\mathcal{L}}}}}}}_{X}X-{{{{{\rm{i}}}}}}{{{{{{\mathcal{L}}}}}}}_{Y}Y\right)\uparrow +\,{{{{{{\mathcal{L}}}}}}}_{Z}Z\downarrow .$$

Here again, the real-valued coefficients ${{{{{{\mathcal{L}}}}}}}_{X},{{{{{{\mathcal{L}}}}}}}_{Y},{{{{{{\mathcal{L}}}}}}}_{{{{{{\rm{Z}}}}}}}$ are found by numerical diagonalization of ${H}_{{LS}}+{H}_{{CF}}$. Writing the full 8-band K.P Hamiltonian including the k-dependent terms, which give rise to dispersion of the band energies away from the zone center (see Eq S19 in Supplementary Note 1), adding the magnetic Hamiltonian given by^42^

8 $${H}_{m}={{-}}\frac{{\mu}_{B}}{{{\hslash}}}\left(3\kappa +1\right){{{{{\bf{L}}}}}}\,{{{{{\boldsymbol{\cdot}}}}}}\,{{{{{\bf{B}}}}}}+{g}_{0}\frac{{\mu}_{B}}{{{\hslash}}}{{{{{\bf{S}}}}}}\,{{{{{\boldsymbol{\cdot}}}}}}\,{{{{{\bf{B}}}}}}{{,}}$$

where ${\mu }_{B}=e\hslash /2{m}_{0}$ is the Bohr magneton, ${g}_{0}=\big|{g}_{e}\big|$ is the free electron-spin *g*-factor ≈ $2.0023$ and $\kappa$ is Luttinger’s magnetic parameter^42^, we evaluate the effective Zeeman Hamiltonian for the lowest conduction band and the valence band using L$\ddot{{{{{{\rm{o}}}}}}}$wdin’s partition method [43], and utilizing the commutators $[{k}_{x},{k}_{y}]=-i\frac{e}{\hslash }{B}_{z}$ and their cyclic permutations [42]. While the g-factors are isotropic in the cubic-phase HOIPs (see Supplementary Note 1, Eq. S26), in tetragonal or orthorhombic perovskites, the symmetry between the orthogonal **x**, **y** and **z** directions is broken, leading to anisotropic g-factors, see the expressions in Supplementary Table 1, and the calculated g-factors shown in Fig. 5. We note that the six principal g-factors (*x, y, z* values for the electron and the hole, respectively) are fully determined by six parameters: these are the bandgap, ${E}_{g}$; the Kane energy, ${E}_{p}$; the spin orbit split-off parameter, $\Delta$; the tetragonal and orthorhombic crystal-field parameters $\delta ,\zeta$ and Luttinger’s magnetic parameter, $\kappa$.

## Supplementary information

Supplementary Information

## Data Availability

All data are available in the main text or the Supplementary Information. Additional data related to the findings of this study may be requested from the authors.

## Code availability

All codes related to the findings of this study may be available upon request.
