# Supplementary material for: Transient quantum beatings of trions in hybrid organic tri-iodine perovskite single crystal
Source: Nat Commun. 2022 Mar 17;13:1428. doi: 10.1038/s41467-022-29053-6 (PMC8931091; doi:10.1038/s41467-022-29053-6)
Supplement: Supplementary file 1 — Supplementary Information [file 41467_2022_29053_MOESM1_ESM.pdf]

Supplemental Information for

Transient quantum beatings of trions in hybrid organic tri-iodine perovskite single crystal

Uyen N. Huynh[1], Ye Liu[2], Ashish Chanana[3], Dipak R. Khanal[1], Peter C. Sercel[4], Jinsong Huang[2], and Z. Valy Vardeny[1,]*

[1] *Department of Physics and Astronomy, University of Utah, Salt Lake City, Utah 84112*
[2] *Department of Applied Physical Sciences, University of North Carolina at Chapel Hill, North Carolina*
[3] *Department of Electrical Engineering, University of Utah, Salt Lake City, Utah 84112*
[4] *Center for Hybrid Organic Inorganic Semiconductors for Energy, Golden, CO, 80401*

Contents

Supplementary Note 1: Description of band structure of $MAPbI_3$ within K.P/crystal field model. 3

Section S1.1: Calculation of the electron and hole g-factors. 7

Section S1.1.1: Electron and hole g-factors in the cubic phase 9

Supplementary Note 2: Exciton fine structure model 9

Section 2.1: Electron-hole exchange........ 11

Section 2.2: Magnetic field splitting of the exciton – Zeeman model 12

Section 2.2: Quantum beating model for magneto-excitons........ 15

Supplementary Note 3: Trion magnetic field splitting and quantum beating 20

Section 3.1: Quantum beating signature of trion 23

Supplementary Note 4: Supplementary data figures. 25

Supplementary References........ 31

List of Supplementary Tables

Supplementary Table 1. Hole and electron g-factors in orthorhombic perovskites. ……….....10

Supplementary Table 2. Energies and transition dipoles for magneto excitons in a magnetic field oriented along the **Z** direction........14

List of Supplementary Figures

Supplementary Fig. 1. Band edge energies in orthorhombic perovskite. 6

Supplementary Fig. 2. Energies and transition oscillator strengths for magneto-excitons in $MAPbI_3$, **B** along [001]. 15

Supplementary Fig. 3. Calculated quantum beating signal for magneto-excitons in MAPbI3 for transient transient photoinduced circular polarized reflection, **B** along [001]............................. 19
Supplementary Fig. 4. Calculated quantum beating signal for magneto-excitons in MAPbI3 for transient photoinduced circular polarized reflection, **B** along [-110]... 20
Supplementary Fig. 5. Creation of a positive trion by resonant optical absorption. 21
Supplementary Fig. 6. Positive trion level splitting in a magnetic field................................... 23
Supplementary Fig. 7. Photoluminescence spectra of a MAPbI3 crystal measured at 10K as a function of pump fluence. 25
Supplementary Fig. 8. Comparison between photoinduced QBs in ‘pristine’and ‘compensated’ MAPbI3 films.. 26
Supplementary Fig. 9. The B-dependent circular-PPR(t) dynamics spin decay constant $\tau(B)$ of positive (fast oscillation) and negative (slow oscillation) trions measured along [100] with **B** along [001]. 27
Supplementary Fig. 10. The *B*-dependent circular-PPR(t) dynamics spin decay constant $\tau(B)$ of positive (fast oscillation) and negative (slow oscillation) trions measured along [110] with **B** along [$1\bar{1}0$]... 28
Supplementary Fig. 11. The spin relaxation lifetime, τ of positive and negative trions (τ_e^+ and τ_h^-, respectively) measured at 4K with **k** along [110] with B along [$1\bar{1}0$]. 29
Supplementary Fig. 12. Temperature dependence of the circular-PPR(t) dynamics and the spin lifetime of positive (τ_e^+) and negative (τ_h^-) trions measured along [100] with **B** along [001]..... 30

Supplementary Note 1: Description of band structure of $MAPbI_3$ within K.P/crystal field model.

In orthorhombic $MAPbI_3$ the conduction and valence band edges are located at the zone center[1] . Within K.P theory the energy band structure near the band edge at Γ is found by writing the Hamiltonian in a basis of the band edge periodic Bloch functions and then diagonalizing the Hamiltonian at non-zero wave vector **k**. Then at any value of **k** the conduction and valence band states may be written approximately in the form,

$$\psi_{n,\boldsymbol{k}}(\mathbf{r}) = u_{n,\boldsymbol{k}=0}(\mathbf{r})\,\frac{1}{\sqrt{V}}e^{i\,\boldsymbol{k}\cdot\boldsymbol{r}}, \qquad \text{(S1)}$$

where n is a band index, $\mathbf{r}$ is the electron coordinate, and V is the crystal volume. In order to describe optical properties we require expressions for the band edge Bloch functions $u_{n,\boldsymbol{k}=0}$ at $\mathbf{k} = 0$ for the conduction and valence bands. We express the Bloch functions within a quasi-cubic model[2], in which the effect of lattice distortion from the cubic phase to the orthorhombic phase is captured in terms of a crystal field. Within this model the valence band functions can be written as the 2-fold degenerate $J = 1/2$, $J_z = \pm 1/2$ states with S orbital symmetry:

$$u_1^v = S\uparrow, \qquad u_2^v = S\downarrow, \qquad \text{(S2)}$$

where the symbol S denotes an orbital function that transforms as an invariant under the operations of the crystal point symmetry group, while ↑ (↓) denote the spin functions with projection $S_z = +1/2\ (-1/2)$. For the conduction band, the Bloch functions have orbital p-symmetry; in a cubic perovskite in the absence of spin-orbit coupling these can be represented by the X, Y, Z orbital basis. To find the Bloch functions in the presence of spin-orbit coupling (SOC) we diagonalize the band-edge Hamiltonian, including the spin-orbit interaction, $H_{LS} = \frac{2}{3}\Delta\,\mathbf{L}\cdot\mathbf{S}$, whose strength is given by Δ, the spin-orbit split-off parameter which separates the upper $J = 3/2$ bands from the lower $J = ½$ conduction bands. We must also include the crystal field (CF) Hamiltonian H_{CF}, which represents the effect of lattice distortions relative to the cubic phase. All together, the band edge Hamiltonian H_{ortho} is,

$$H_{ortho} = H_0 + H_{CF} + H_{LS}\,. \qquad \text{(S3)}$$

Here, H_0 gives the band edge energies in the absence of SOC and CF splitting. The crystal field Hamiltonian H_{CF} can be expressed in invariant form as [2],

$$H_{CF} = \left(\zeta - \frac{\delta}{3}\right) L_x^2 + \left(-\zeta - \frac{\delta}{3}\right) L_y^2 + \left(\frac{2}{3}\delta\right) L_z^2, \qquad \text{(S4)}$$

where L_x, L_y, L_z are the *x*,*y*,*z* components of the orbital angular momentum operator, and the crystal field parameters δ, ζ reflect symmetry breaking relative to the cubic phase in the **z** and the **x**, **y** directions, respectively; we call δ, ζ the "tetragonal" and "orthorhombic crystal fields, respectively. The portion of the Hamiltonian H_{ortho} given by $H_0 + H_{CF}$ is represented in the S, X, Y, Z orbital basis within a quasi-cubic approximation by [2],

$$\widetilde{H}_0 + \widetilde{H}_{CF} = \begin{pmatrix} E_v^0 & 0 & 0 & 0 \\ 0 & E_c^0 + \frac{\delta}{3} - \zeta & 0 & 0 \\ 0 & 0 & E_c^0 + \frac{\delta}{3} + \zeta & 0 \\ 0 & 0 & 0 & E_c^0 - \frac{2}{3}\delta \end{pmatrix}, \tag{S5}$$

where E_v^0 and E_c^0 are valence and conduction band edges without SOC or CF splitting. The eigenvectors are found by diagonalizing the matrix $\widetilde{H}_{ortho}$ in Eq. S3. Within the basis of eigenstates of total angular momentum, $J = L + S$, taken in the order $J = 1/2\ (L = 0)$, $J = 1/2\ (L = 1)$, $J = 3/2\ (L = 1)$, with the values of J_z for each J running over $J \geq J_z \geq -J$ ordered high to low, this has the matrix representation,

$$\widetilde{H}_{ortho} = \begin{pmatrix} E_v^0 & 0 & 0 & 0 & 0 & 0 & 0 & 0 \\ 0 & E_v^0 & 0 & 0 & 0 & 0 & 0 & 0 \\ 0 & 0 & E_c^0 & 0 & 0 & \frac{\sqrt{2}}{3}\delta & 0 & \sqrt{\frac{2}{3}}\zeta \\ 0 & 0 & 0 & E_c^0 & -\sqrt{\frac{2}{3}}\zeta & 0 & -\frac{\sqrt{2}}{3}\delta & 0 \\ 0 & 0 & 0 & -\sqrt{\frac{2}{3}}\zeta & E_c^0 + \Delta + \frac{\delta}{3} & 0 & \frac{\zeta}{\sqrt{3}} & 0 \\ 0 & 0 & \frac{\sqrt{2}}{3}\delta & 0 & 0 & E_c^0 + \Delta - \frac{\delta}{3} & 0 & \frac{\zeta}{\sqrt{3}} \\ 0 & 0 & 0 & -\frac{\sqrt{2}}{3}\delta & \frac{\zeta}{\sqrt{3}} & 0 & E_c^0 + \Delta - \frac{\delta}{3} & 0 \\ 0 & 0 & \sqrt{\frac{2}{3}}\zeta & 0 & 0 & \frac{\zeta}{\sqrt{3}} & 0 & E_c^0 + \Delta + \frac{\delta}{3} \end{pmatrix}, \tag{S6}$$

The conduction band edge states can be solved exactly in the tetragonal case, when $\delta \neq 0$ with vanishing orthorhombic crystal field, $\zeta = 0$. The lowest conduction band in this case has energy [2-4],

$$E_c(\Delta, \delta) = \mathrm{E}_c^0 + \frac{3\Delta - \delta}{6} - \frac{1}{2}\sqrt{\delta^2 - \frac{2}{3}\delta\Delta + \Delta^2}; \left(J_z = \pm\frac{1}{2}\right). \tag{S7}$$

Note that the energy zero has been set so that with zero crystal field the conduction band edge is at energy $E_c = E_c^0$. The upper conduction bands, which originate from the $J = 3/2$ states of the cubic crystal, are split by the tetragonal crystal field. In the tetragonal case these bands have band-edge energies given by [2-4],

$$E_{le}(\Delta,\delta) = E_c^0 + \frac{3\Delta - \delta}{6} + \frac{1}{2}\sqrt{\delta^2 - \frac{2}{3}\delta\Delta + \Delta^2}; \left(J_z = \pm\frac{1}{2}\right) \qquad \text{(S8)}$$

$$E_{he}(\Delta,\delta) = E_c^0 + \frac{\delta}{3} + \Delta; \qquad \left(J_z = \pm\frac{3}{2}\right) \qquad \text{(S9)}$$

Finding a closed form expression for the band edge energies and Bloch functions in the presence of an orthorhombic crystal field is more complicated since the energy is a cubic function the orthorhombic crystal field. However the band edge energies and eigenvectors can be readily found by numerical diagonalization. An approximate result for the lowest band edge energy is [2],

$$E_c(\Delta,\delta,\zeta) \approx \frac{\mathrm{E_c}(\Delta,\delta) + \mathrm{E_{he}}(\Delta,\delta)}{2} + \frac{1}{2}\sqrt{(E_{he}(\Delta,\delta) - \mathrm{E_c}(\Delta,\delta))^2 + 4\zeta^2\cos^2\theta}\ , \qquad \text{(S10)}$$

where the phase angle θ is given in terms of the spin orbit coupling, Δ and the tetragonal crystal field δ by [3,4]:

$$\tan 2\theta = \frac{2\sqrt{2}\ \Delta}{\Delta - 3\delta}, \qquad \theta \le \frac{\pi}{2}, \qquad \text{(S11)}$$

while the lowest energy conduction bands have band edge Bloch functions that can be expressed in the general form,

$$u_1^{\mathcal{C}} = -\,\mathcal{C}_Z\, Z \uparrow - (\mathcal{C}_X X + i\mathcal{C}_Y Y) \downarrow,$$

$$u_2^{\mathcal{C}} = -(\mathcal{C}_X X - i\,\mathcal{C}_Y Y) \uparrow + \mathcal{C}_Z Z \downarrow. \qquad \text{(S12)}$$

In these expression the symbols X, Y, Z denote orbital functions that transform like x ,y, z under rotations, while $\mathcal{C}_X, \mathcal{C}_Y, \mathcal{C}_\mathrm{Z}$ are c-numbers that reflect the effect of the crystal field splitting. The coefficients $\mathcal{C}_X, \mathcal{C}_Y, \mathcal{C}_\mathrm{Z}$ can be written approximately in terms of two phase angles, θ and ϕ, determined by the crystal fields, δ, and ζ, as[2],

$$\mathcal{C}_X \approx \mathcal{C}_X(\theta,\varphi) = \frac{\cos\phi\cos\theta - \sin\phi}{\sqrt{2}}\ ,$$

$$\mathcal{C}_Y \approx \mathcal{C}_Y(\theta,\varphi) = \frac{\cos\phi\cos\theta + \sin\phi}{\sqrt{2}}\ ,$$

$$\mathcal{C}_Z \approx \mathcal{C}_Z(\theta,\varphi) = \cos\phi\sin\theta\ . \qquad \text{(S13)}$$

In these expressions, the phase angle θ is given in terms of the spin orbit coupling, Δ and the tetragonal crystal field δ by Eq. S11while the phase angle φ has non-zero values in the orthorhombic structure and is determined by[2] ,

$$\tan 2\varphi = \frac{-4\,\zeta\,\cos\theta}{\Delta + \delta + \sqrt{\Delta^2 - \frac{2}{3}\Delta\,\delta + \delta^2}}\ . \qquad \text{(S14)}$$

In applications in this work, the coefficients $\mathcal{C}_X, \mathcal{C}_Y, \mathcal{C}_\mathrm{Z}$ were determined numerically as a function of the crystal field parameters δ, ζ and the SOC parameter Δ by diagonalizing the full Hamiltonian Eq. S6 rather than using approximate Eq. S13, in order to ensure accuracy. The energies of the

heavy- and light-electron band edges can also written in approximate closed form, but in this work we find the energies by numerical diagonalization of Eq. S6. The corresponding Bloch functions can be shown to have the following general forms: For the heavy electron band,

$$u_1^{\mathcal{H}} = -(\mathcal{H}_X X + \mathrm{i}\mathcal{H}_Y Y)\uparrow + \mathcal{H}_Z Z\downarrow,$$
$$u_2^{\mathcal{H}} = \mathcal{H}_Z Z\uparrow + (\mathcal{H}_X X - \mathrm{i}\,\mathcal{H}_Y Y)\downarrow, \qquad \text{(S15)}$$

where the coefficients $\mathcal{H}_X, \mathcal{H}_Y, \mathcal{H}_Z$ are found numerically as functions of δ, ζ and Δ. The corresponding result for the light-electron band is,

$$u_1^{\mathcal{L}} = \mathcal{L}_Z Z\uparrow - (\mathcal{L}_X X + \mathrm{i}\mathcal{L}_Y Y)\downarrow,$$
$$u_2^{\mathcal{L}} = (\mathcal{L}_X X - \mathrm{i}\,\mathcal{L}_Y Y)\uparrow + \mathcal{L}_Z Z\downarrow. \qquad \text{(S16)}$$

Here again, the coefficients $\mathcal{L}_X, \mathcal{L}_Y, \mathcal{L}_Z$ are found numerically.

Supplementary Fig. 1 shows the calculated conduction band edge energies for the light-, heavy- and conduction electron bands computed by diagonalization of Eq. S6, parametrically as a function of the orthorhombic crystal field, ζ, with fixed SOC split off parameter Δ =1400meV [4] and tetragonal crystal field δ = + 349.8 meV, determined by fitting to the measured electron and hole g-factors as described in the main text. Note that the tetragonal crystal field is positive, in agreement with density functional theory calculations of band-structure of tetragonal $MAPbI_3$ reported in Refs.[1,5], the hybrid density function theory calculations for both tetragonal and orthorhombic $MAPbI_3$ reported in Ref. [2], and the 16-band K.P model for tetragonal $MAPbI_3$ reported in Ref.[6].

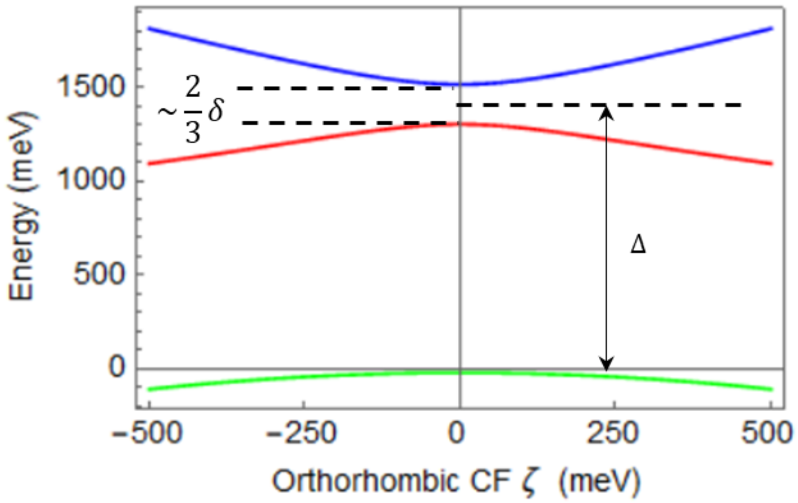

Supplementary Fig. 1. Band edge energies in orthorhombic perovskite. The figure shows calculated conduction band edge energies for the light-, heavy- and conduction electron bands computed by diagonalizing Eq S3 parametrically as a function of the orthorhombic crystal field, $\boldsymbol{\zeta}$, with fixed SOC split off parameter $\boldsymbol{\Delta}$ =1.4 eV[4] and tetragonal crystal field $\boldsymbol{\delta}$ = + 349.8 meV, determined by fitting to the measured electron and hole g-factors as described in the main text. The splitting between the heavy- and light- electron band edges for zero $\boldsymbol{\zeta}$ is approximately $\mathbf{2/3}\ \boldsymbol{\delta}$. The legends labels he, le and ce stand for heavy-electron, light-electron, and conduction-electron bands, respectively.

Section S1.1: Calculation of the electron and hole g-factors.

Using the results in the last section for the band edge energies and Bloch functions in the orthorhombic crystal structure, we can apply the K.P theory to calculate the electron and hole g-factors. To do we first write the full K.P Hamiltonian reflecting coupling between the conduction and valence bands near the zone center Γ point, the conduction band Hamiltonian H_0 can be written as,

$$H^0 = H_{\mathrm{k}} + H_{CF} + H_{LS}\,, \qquad \text{(S17)}$$

where H_{k} represents the band edge energies in the absence of SOC and CF splitting plus the k-dependent terms which give rise to dispersion of the band energies away from the zone center; while $H_{LS} = \frac{2}{3}\Delta\,\mathbf{L}\cdot\mathbf{S}$ is the spin orbit coupling and H_{CF} is the crystal field Hamiltonian given in Eq. S4-S5 apart from the band edge energies E_v^0, E_c^0. The Hamiltonian H_k for the cubic phase has the following representation in a basis of states S,X,Y,Z in the absence of spin orbit coupling: $\widetilde{H}(\boldsymbol{k}) =$

$$\begin{pmatrix} E_v^0 & i\frac{\hbar}{m_0}k_xP & i\frac{\hbar}{m_0}k_yP & i\frac{\hbar}{m_0}k_zP \\ -i\frac{\hbar}{m_0}k_xP & E_c^0+\frac{\hbar^2}{2m_0}\left(A\mathrm{k}_x{}^2+B\left(\mathrm{k}_y{}^2+\mathrm{k}_z{}^2\right)\right) & \frac{\hbar^2}{2m_0}C\,k_xk_y & \frac{\hbar^2}{2m_0}C\,k_xk_z \\ -i\frac{\hbar}{m_0}k_yP & \frac{\hbar^2}{2m_0}C\,k_xk_y & E_c^0+\frac{\hbar^2}{2m_0}\left(A\mathrm{k}_x{}^2+B\left(\mathrm{k}_y{}^2+\mathrm{k}_z{}^2\right)\right) & \frac{\hbar^2}{2m_0}C\,k_yk_z \\ -i\frac{\hbar}{m_0}k_zP & \frac{\hbar^2}{2m_0}C\,k_xk_z & \frac{\hbar^2}{2m_0}C\,k_yk_z & E_c^0+\frac{\hbar^2}{2m_0}\left(A\mathrm{k}_x{}^2+B\left(\mathrm{k}_y{}^2+\mathrm{k}_z{}^2\right)\right) \end{pmatrix}.$$

(S18)

Here $P = -i\,\langle S|\hat{p}_x|X\rangle = -i\,\langle S|\hat{p}_y|Y\rangle = -i\,\langle S|\hat{p}_z|Z\rangle$ is the Kane momentum matrix element[7] . Following Luttinger[8] the terms A, B, C can be expressed in terms of the well-known Luttinger mass parameters as follows:

$$A = \gamma_1 + 4\,\gamma_2,$$

$$B = \gamma_1 - 2\,\gamma_2\,,$$

$$C = 6\,\gamma_3\;. \qquad \text{(S19)}$$

Note that the quasi-cubic treatment here assumes that the effect of symmetry breaking from the cubic phase is due solely to the effect of the crystal field, i.e., the Kane and Luttinger parameters have the same symmetry relations as in the cubic phase. Our treatment differs in this sense from that in Ref.[4], where for the tetragonal phase, separate Kane and Luttinger parameters are introduced for the directions parallel and perpendicular to the tetragonal c-axis, but this approach leads to a proliferation of undetermined parameters.

Introducing spin orbit coupling as before, the 8x8 Hamiltonian $H_{\mathrm{k}} + H_{LS}$ is diagonalized at $\mathbf{k} = 0$ in a basis of eigenstates of total angular momentum, $\mathbf{J} = \mathbf{L} + \mathbf{S}$. Adding the crystal field Hamiltonian, as in the last section, the total Hamiltonian is diagonalized using Bloch functions Eq. S2 for the valence bands, Eq. S11 for the conduction bands and Eqs. S14, S15 for the heavy- and light- electron bands respectively. The matrix representation of the Hamiltonian can be written in closed form in this basis but we will not show it here; it is represented as an 8x8 matrix.

With the Hamiltonian for the orthorhombic crystal determined, we can calculate the electron and hole g-factors by considering the effect of an externally applied magnetic field. The magnetic Hamiltonian is given by[8],

$$H_m = -\frac{\mu_b}{\hbar}(3\kappa+1)\ \mathbf{L}\cdot\mathbf{B}\ + g_0\frac{\mu_b}{\hbar}\ \mathbf{S}\cdot\ \mathbf{B}\ , \tag{S20}$$

where $\mu_b = e\hbar/2m_0$ is the Bohr magneton, $g_0 = |g_e|$ is the free electron spin g-factor, approximately equal to 2.0023, and κ is Luttinger's magnetic parameter[8]. We evaluate the effective Zeeman Hamiltonian for the lowest conduction band and the valence band using Löwdin's partition method[9]. The effective magnetic Hamiltonian has the form for electrons (holes) given by,

$$H_{Z,e(h)}^{eff} = \frac{\mu_b}{\hbar}\left\{g_{e(h),x}J_{e(h),x}\,B_x + g_{e(h),y}\,J_{e(h),y}\,B_y + g_{e(h),z}\,J_{e(h),z}\,B_z\right\}, \tag{S21}$$

which is represented in the band edge basis as a 2x2 matrix. We find the g-factors using Löwdin's method[9] for constructing the effective Hamiltonian from the 8x8 K.P Hamiltonian[4] :

$$\widetilde{H}_{ij}^{eff}(\mathbf{k}) = \widetilde{H}_{ij}(\mathbf{k})\ +\ \sum_n \frac{\widetilde{H}_{in}(\mathbf{k})\,\widetilde{H}_{nj}(\mathbf{k})}{E_i(\mathbf{k})\ -\ \widetilde{H}_{nn}}. \tag{S22}$$

We apply this procedure using the 8x8 Hamiltonian derived from Eq. S17 plus the magnetic field Hamiltonian Eq. S20, approximate the energies $E_i(\mathbf{k})$ and the matrix elements $\widetilde{H}_{nn}$ by the band edge values; and utilize the commutators $[k_x, k_y] = -i\,\frac{e}{\hbar}\,B_z$ and its cyclic permutations[8]. We retain only those terms linear in the applied magnetic field. Eliminating the Kane momentum matrix element in favor of the Kane energy, defined by,

$$E_p = \frac{2\,P^2}{m_0}, \tag{S23}$$

We obtain the electron and hole g-factors after a straightforward but lengthy calculation. For the holes, the g-factor components are,

$$g_{h,x} = g_0 - 2E_p\left(\frac{\mathcal{C}_Y\,\mathcal{C}_Z}{E_c - E_v} - \frac{\mathcal{L}_Y\,\mathcal{L}_Z}{E_{le} - E_v} - \frac{\mathcal{H}_Y\,\mathcal{H}_Z}{E_{he} - E_v}\right),$$

$$g_{h,y} = g_0 - 2E_p\left(\frac{\mathcal{C}_X\mathcal{C}_Z}{E_c - E_v} - \frac{\mathcal{L}_X\,\mathcal{L}_Z}{E_{le} - E_v} + \frac{\mathcal{H}_X\,\mathcal{H}_Z}{E_{he} - E_v}\right),$$

$$g_{h,z} = g_0 - 2\,E_p\left(\frac{\mathcal{C}_X\,\mathcal{C}_Y}{E_c - E_v} + \frac{\mathcal{L}_X\,\mathcal{L}_Y}{E_{le} - E_v} - \frac{\mathcal{H}_X\,\mathcal{H}_Y}{E_{he} - E_v}\right). \tag{S24}$$

Here, the band edge energies $E_c, E_{he},$ and E_{le} are found by numerical diagonalization and E_v is determined from the measured the bandgap via $E_\mathrm{v} = E_c(\Delta, \delta, \zeta) - E_\mathrm{g}$. The coefficients $\mathcal{C}_i$, $\mathcal{H}_i$ and $\mathcal{L}_i$ that enter this expression are determined numerically as well. We note that hole g-factor is the same in both the electron and hole representations as described in detail in Ref. [10]. The electron g-factors are found as,

$$g_{e,x} = 2\,\mathcal{C}_Y\,\mathcal{C}_Z\,\frac{E_p}{E_g} - \left\{4\,\mathcal{C}_Y\,\mathcal{C}_Z\,(3\,\kappa_2 + 1) - g_0\left({\mathcal{C}_X}^2 - {\mathcal{C}_Y}^2 - {\mathcal{C}_Z}^2\right)\right\}$$

$$g_{e,y} = 2\, \mathcal{C}_X \mathcal{C}_Z \frac{E_p}{E_g} - \{4\, \mathcal{C}_X \mathcal{C}_Z (3\, \kappa_2 + 1) - \; g_0 ({\mathcal{C}_Y}^2 - {\mathcal{C}_X}^2 - {\mathcal{C}_Z}^2)\}$$

$$g_{e,z} = 2\, \mathcal{C}_X \mathcal{C}_Y \frac{E_p}{E_g} - \{4\, \mathcal{C}_X \mathcal{C}_Y (3\, \kappa_2 + 1) - \; g_0 ({\mathcal{C}_Z}^2 - {\mathcal{C}_X}^2 - {\mathcal{C}_Y}^2)\} \quad \text{(S25)}$$

We verified that the expressions in Eqs. S24-S25 reproduce previously derived results[4] for the tetragonal and cubic phases in the corresponding limits.

We note that the six g-factors (*x, y, z* values for the electron and the hole, respectively) are fully determined by six parameters: These are the band gap, E_g; the Kane energy, E_p; the spin orbit split-off parameter, Δ; the tetragonal and orthorhombic crystal field parameters δ, ζ, and Luttinger's magnetic parameter, κ. In practice the bandgap is easily measured; the split-off parameter can be measured or calculated[4], so that the remaining parameters E_p, δ, ζ and κ can be determined by measuring the electron and hole g-factors along two symmetry axes of the crystal as described in the main text. The expressions are summarized in Supplementary Table 1.

Section S1.1.1: Electron and hole g-factors in the cubic phase

It is a useful to evaluate the expressions derived above for the orthorhombic crystal phase against the limit of cubic crystal symmetry, that is, when the crystal fields $\delta = \zeta = 0$. In this case, the lowest conduction bands have band-edge Bloch functions that can be represented as eigenstates of the total angular momentum, $\mathbf{J} = \mathbf{L} + \mathbf{S}$, with $J = 1/2$, and the coefficients in Eq (S12) can be shown to take the values, $\mathcal{C}_X = \mathcal{C}_Y = \mathcal{C}_Z = 1/\sqrt{3}$. Similarly, the upper conduction bands become 4-fold degenerate, separated in energy by Δ from the lower $J = 1/2$ conduction bands, and have band-edge Bloch functions that can be represented as eigenstates of $\mathbf{J} = \mathbf{L} + \mathbf{S}$ with $J = 3/2$. Then, the coefficients in Eqs. S15, S16 take the values, $\mathcal{H}_X = \mathcal{H}_Y = 1/\sqrt{2}$; $\mathcal{H}_Z = 0$, and $\mathcal{L}_X = \mathcal{L}_Y = 1/\sqrt{6}$; $\mathcal{L}_Z = \sqrt{2/3}$. Using these coefficients leads to the following expressions for the isotropic electron and hole g-factors in the cubic crystal structure:

$$g_h^{cubic} = g_0 - \frac{2}{3} E_p \left(\frac{1}{E_g} - \frac{1}{E_g + \Delta} \right); \; g_e^{cubic} = \frac{2}{3} \frac{E_p}{E_g} - (4\, \kappa_1 + 2), \quad \text{(S26)}$$

Supplementary Note 2: Exciton fine structure model

In this section we describe the Hamiltonian that determines the exciton fine structure in orthorhombic perovskite crystals such as $MAPbI_3$. We consider the effects of the short-range (SR) electron-hole exchange interaction, H_{SR}, and the effects of an applied magnetic field, **B**, through the Zeeman Hamiltonian H_Z. The magneto-exciton Hamiltonian is thus,

$$H = H_{SR} + H_Z \quad \text{(S27)}$$

We will determine the eigenstates of this Hamiltonian for free excitons in orthorhombic $MAPbI_3$. We write the exciton state as the product wave function[11],

$$\psi^{ex}_{i,j;\mathbf{K};nlm}(\mathbf{r}_e, \mathbf{r}_h) = u_i^e(\mathbf{r}_e)\; u_j^h(\mathbf{r}_h)\; f_{\mathbf{K};nlm}(\mathbf{r}_e, \mathbf{r}_h). \quad \text{(S28)}$$

Supplementary Table 1. Hole and electron g-factors in orthorhombic perovskites.

The subscripts x, y, z denote the [1,0,0], [0,1,0] and [0,0,1] crystallographic directions. The coefficients $\mathcal{C}_X, \mathcal{C}_Y, \mathcal{C}_Z$; $\mathcal{L}_X, \mathcal{L}_Y, \mathcal{L}_Z$; and $\mathcal{H}_X, \mathcal{H}_Y, \mathcal{H}_Z$, determine the lowest conduction band Bloch functions, and the light- and heavy-electron band Bloch functions. These are determined by numerical diagonalization of the 8-band K.P Hamiltonian, Eq. S6, which includes the tetragonal and orthorhombic crystal fields δ and ζ, and the SOC parameter Δ. In the expressions, $g_0 \approx$ 2.0023 is the free electron spin g-factor, $E_\mathrm{v} = E_c(\Delta, \delta, \zeta) - E_\mathrm{g}$, where E_g is the bandgap, E_p is the Kane energy, and κ is Luttinger's magnetic parameter; Δ is the SOC split-off parameter.

Hole g-factors	Electron g-factors
$g_X^h = g_0 - 2E_p\left(\frac{\mathcal{C}_Y\,\mathcal{C}_Z}{E_c - E_v} - \frac{\mathcal{L}_Y\,\mathcal{L}_Z}{E_{le} - E_v} - \frac{\mathcal{H}_Y\,\mathcal{H}_Z}{E_{he} - E_v}\right)$	$g_X^e = 2\,\mathcal{C}_Y\,\mathcal{C}_Z\,\frac{E_p}{E_g} - \{4\,\mathcal{C}_Y\,\mathcal{C}_Z\,(3\,\kappa_2 + 1) - g_0(\mathcal{C}_X{}^2 - \mathcal{C}_Y{}^2 - \mathcal{C}_Z{}^2)\}$
$g_Y^h = g_0 - 2E_p\left(\frac{\mathcal{C}_X\mathcal{C}_Z}{E_c - E_v} - \frac{\mathcal{L}_X\,\mathcal{L}_Z}{E_{le} - E_v} + \frac{\mathcal{H}_X\,\mathcal{H}_Z}{E_{he} - E_v}\right)$	$g_Y^e = 2\,\mathcal{C}_X\mathcal{C}_Z\frac{E_p}{E_g} - \{4\,\mathcal{C}_X\mathcal{C}_Z(3\,\kappa_2 + 1) - g_0(\mathcal{C}_Y{}^2 - \mathcal{C}_X{}^2 - \mathcal{C}_Z{}^2)\}$
$g_Z^h = g_0 - 2\,E_p\left(\frac{\mathcal{C}_X\,\mathcal{C}_Y}{E_c - E_v} + \frac{\mathcal{L}_X\,\mathcal{L}_Y}{E_{le} - E_v} - \frac{\mathcal{H}_X\,\mathcal{H}_Y}{E_{he} - E_v}\right)$	$g_Z^e = 2\,\mathcal{C}_X\mathcal{C}_Y\frac{E_p}{E_g} - \{4\,\mathcal{C}_X\mathcal{C}_Y(3\,\kappa_2 + 1) - g_0(\mathcal{C}_Z{}^2 - \mathcal{C}_X{}^2 - \mathcal{C}_Y{}^2)\}$

This is a product of the band-edge periodic basis functions for the electron and hole, u_i^e and u_j^h, respectively, which contain information pertaining to the spin of the exciton, and the envelope function for the exciton, $f_{n,K}(\mathbf{r}_e, \mathbf{r}_h)$, which describes the relative and center of mass (COM) motion of the electron and hole in the exciton state. For free excitons, the envelope function is specified in terms of the COM wave vector $\mathbf{K}$ and quantum numbers n, l, m associated with the state of internal relative motion of the electron and hole, which we describe in terms of a hydrogenic model. The lowest exciton is a 1s hydrogenic state with $n = 1$ and $l = 0$ so that,

$$f_{\mathbf{K};1s}\,(\mathbf{r_e}, \mathbf{r}_\mathrm{h}) = \frac{1}{\sqrt{V}}\,e^{i\mathbf{K}\cdot\mathbf{R}}\phi_{1s}(\mathbf{r_e} - \mathbf{r}_\mathrm{h}), \tag{S29}$$

where the wave function is the relative coordinate is given by,

$$\phi_{1s}(r) = \frac{2}{a_x{}^{3/2}}\,e^{-r/a_x}\,\frac{1}{\sqrt{4\pi}}\ \ . \tag{S30}$$

The Bloch functions for the hole and the electron, u_h and u_e are given in Eqs. S2 and S12 respectively. Using these expressions we form a Bloch function basis of electron hole pair states:

$$P_1 = u_1^e u_1^h; \quad P_2 = u_1^e u_2^h; \quad P_3 = u_2^e u_1^h; \; P_4 = u_2^e u_2^h \tag{S31}$$

With the wavefunction in hand, we start our analysis by developing the electron-hole exchange Hamiltonian.

Section 2.1: Electron-hole exchange

The short range exchange interaction can be written as an effective spin operator for the bulk exciton which can be written[11],

$$H_{SR} = \frac{1}{2} C\, \Theta\, [\mathbf{I} - \boldsymbol{\sigma}_{\mathrm{e}}.\boldsymbol{\sigma}_{\mathrm{h}}]\,. \tag{S32}$$

Here, $\boldsymbol{\sigma}_{\mathrm{e}}$ and $\boldsymbol{\sigma}_{\mathrm{h}}$ are Pauli operators representing the true spin (not the total angular momentum) of the electron and the hole, respectively; $\mathbf{r}_{\mathrm{e}}$ and $\mathbf{r}_{\mathrm{h}}$ are their respective position vectors, C is the exchange constant for the material, and Ω is the unit cell volume. The term Θ is the electron-hole overlap factor, representing the probability that the electron and hole reside in the same unit cell. For the exciton $n, \mathbf{K}$ it can be written,

$$\Theta_{n,\mathbf{K}} = \Omega \iint_V d^3\mathbf{r}_{\mathrm{e}} d^3\,\mathbf{r}_{\mathrm{h}}\, f^*_{n,K}(\mathbf{r}_{\mathrm{e}}, \mathbf{r}_{\mathrm{h}}) \delta(\mathbf{r}_{\mathrm{e}} - \mathbf{r}_{\mathrm{h}}) f_{n,\boldsymbol{K}}(\mathbf{r}_{\mathrm{e}}, \mathbf{r}_{\mathrm{h}})$$

$$= \Omega \int_V d^3\mathbf{r}\; | f_{n,\mathbf{K}}(\mathbf{r}, \mathbf{r})|^2. \tag{S33}$$

The integration above is taken over the entire volume, V, of the crystal. The "ground state" exciton, by which we mean the lowest energy exciton state for a given wave vector $\mathbf{K}$, corresponds to the 1s hydrogenic state of relative motion as discussed above. Using Eqs. S29-S30 for this state, the overlap factor is,

$$\Theta_{1s} = \frac{\Omega}{\pi a_x^3}\;, \tag{S34}$$

where a_x is the exciton Bohr radius. With this result we can rewrite Eq. S32 for the ground exciton in terms of the singlet triplet splitting $\hbar\omega_{st} = 2/3\; C\Theta_{1s}$,

$$H_{SR} = \frac{3}{4}\, \hbar\omega_{st}\, [\mathbf{I} - \boldsymbol{\sigma}_{\mathrm{e}}.\boldsymbol{\sigma}_{\mathrm{h}}]. \tag{S35}$$

This equation is our effective spin operator for the electron hole exchange interaction. With the eh pair basis Eq S31, we find the exchange Hamiltonian has the following representation:

$$\widetilde{H}_{Pair} = \frac{3}{2}\, \hbar\omega_{st} \times \begin{pmatrix} \mathcal{C}_x^2 + \mathcal{C}_y^2 & 0 & 0 & \mathcal{C}_y^2 - \mathcal{C}_x^2 \\ 0 & \mathcal{C}_z^2 & \mathcal{C}_z^2 & 0 \\ 0 & \mathcal{C}_z^2 & \mathcal{C}_z^2 & 0 \\ \mathcal{C}_y^2 - \mathcal{C}_x^2 & 0 & 0 & \mathcal{C}_x^2 + \mathcal{C}_y^2 \end{pmatrix}. \tag{S36}$$

It is convenient to diagonalize this Hamiltonian with the transformation[12],

$$\widetilde{H}_{XYZ} = \widetilde{M}_2{}^{\dagger} \widetilde{M}_1{}^{\dagger} \widetilde{H}_{Pair}\; \widetilde{M}_1\, \widetilde{M}_2$$

Where the unitary transformation matrices $\widetilde{M}_1$, $\widetilde{M}_2$ are given by,

$$\widetilde{M}_1 = \begin{pmatrix} 0 & 1 & 0 & 0 \\ \frac{-1}{\sqrt{2}} & 0 & \frac{1}{\sqrt{2}} & 0 \\ \frac{1}{\sqrt{2}} & 0 & \frac{1}{\sqrt{2}} & 0 \\ 0 & 0 & 0 & 1 \end{pmatrix} \quad \text{and} \quad \widetilde{M}_2 = \begin{pmatrix} 1 & 0 & 0 & 0 \\ 0 & \frac{-1}{\sqrt{2}} & \frac{i}{\sqrt{2}} & 0 \\ 0 & 0 & 0 & 1 \\ 0 & \frac{1}{\sqrt{2}} & \frac{i}{\sqrt{2}} & 0 \end{pmatrix} .$$

The first transformation ($\widetilde{M}_1$) transforms the Hamiltonian to a basis of total angular momentum $F = J_e + J_h$, taken in the order, $|F, F_z\rangle = |0,0\rangle, |1,1\rangle, |1,0\rangle, |1,-1\rangle$, while the second diagonalizes the Hamiltonian in a basis of exciton states $|u_{X_i}\rangle$, taken in the order $|D\rangle, |X\rangle, |Y\rangle, |Z\rangle$, whose transition dipoles to the crystal ground state respectively vanish (D) or are aligned along the symmetry directions **X**, **Y**, **Z** which correspond to the three mutually orthogonal C_2 symmetry axes of the orthorhombic crystal system. In this basis the exchange Hamiltonian is given by,

$$\widetilde{H}_{DXYZ} = \begin{pmatrix} E_D & 0 & 0 & 0 \\ 0 & E_X & 0 & 0 \\ 0 & 0 & E_Y & 0 \\ 0 & 0 & 0 & E_Z \end{pmatrix}. \tag{S37}$$

In this expression, the exciton eigen-energies are given by[2],

$$E_D = 0 \ ,$$

$$E_X = 3\hbar\omega_{st}\, C_x^2 \ ,$$

$$E_Y = 3\hbar\omega_{st}\ C_y^2 \ ,$$

$$E_Z = 3\ \hbar\omega_{st}\ C_z^2 \ . \tag{S38}$$

The corresponding dipole transition moments, $\langle u_{X_i}|\mathbf{P}|G\rangle$, evaluated between the Bloch functions of each state, $|u_{X_i}\rangle$ and the $|G\rangle$ is the crystal ground state, are given by[2],

$$\tilde{\mathbf{p}} = 0 \ ,$$

$$\tilde{\mathbf{p}}_X = \sqrt{2}\, C_x\, P\, \hat{x} \ ,$$

$$\tilde{\mathbf{p}}_Y = \sqrt{2}\, C_y\, P\, \hat{y} \ ,$$

$$\tilde{\mathbf{p}}_Z = \sqrt{2}\, C_z\, P\, \hat{z} \ . \tag{S39}$$

where $P = |\langle S|\mathbf{P}|Z\rangle|$ is the Kane momentum matrix element that appeared above.

In Ref. [6] the exchange constant and exciton radius are given for orthorhombic MAPI as $\hbar\omega_{st} =$ 0.126 meV and $a_x = 4.8$nm. In our model, we use these parameters, and determine the crystal fields, δ and ζ, and the spin orbit coupling split-off parameter, Δ, by fitting the measured g-factors of the electron and hole using the orthorhombic model presented above. This determines the phase angles and hence the phase angles θ, φ.

Section 2.2: Magnetic field splitting of the exciton – Zeeman model

The Zeeman Hamiltonian for the exciton, neglecting crystal anisotropy, can be written [4,10] ,

$$H_M = g_e\mu_B\, \mathbf{J_e} \cdot \mathbf{B} + g_h\mu_B\, \mathbf{J_h} \cdot \mathbf{B}\,, \tag{S40}$$

where, $\mathbf{J_e}, \mathbf{J_h}$ are the vector total angular momentum operators for the electron and hole, respectively, μ_B is the Bohr magneton, and g_e, g_h are the electron and hole g-factors. Note that the hole term above is written with a positive sign, moreover the magnitude and the sign of the hole g-factor is the same in both the electron and hole representations as noted in Ref. [10]. The Zeeman Hamiltonian expression is easily generalized to the case of orthorhombic symmetry,

$$H_M = \mu_B \{ (g_{e,x} J_{e,x} + g_{h,x} J_{hx}) B_x + (g_{e,y} J_{e,y} + g_{h,y} J_{hy}) B_y + (g_{e,z} J_{e,z} + g_{h,z} J_{h,z}) B_z \} \tag{S41}$$

Expressing the Hamiltonian in the pair basis Eq. S31 and then transforming to the exciton basis D,X,Y,Z as before, we find the representation of the Zeeman Hamiltonian as,

$$\widetilde{H}_{DXYZ} = \frac{\mu_b}{2} \begin{pmatrix} 0 & B_x(g_{h,x} - g_{e,x}) & B_y(g_{h,y} - g_{e,y}) & B_z(g_{h,z} - g_{e,z}) \\ B_x(g_{h,x} - g_{e,x}) & 0 & -iB_z(g_{e,z} + g_{h,z}) & +i\,B_y(g_{e,y} + g_{h,y}) \\ B_y(g_{h,y} - g_{e,y}) & +iB_z(g_{e,z} + g_{h,z}) & 0 & -i\,B_x(g_{e,x} + g_{h,x}) \\ B_z(g_{h,z} - g_{e,z}) & -i\,B_y(g_{e,y} + g_{h,y}) & +iB_x(g_{e,x} + g_{h,x}) & 0 \end{pmatrix}. \tag{S42}$$

Inspection of this matrix indicates that in the presence of a magnetic field the dark exciton state D will mix with any bright exciton whose transition dipole has a non-zero projection along the magnetic field. If the magnetic field is along the **Z** direction, the states D and Z will mix; if **B** is along **X**, then the states D and X will mix. At the same time, a magnetic field component i will mix states $j \neq i$ and $k \neq i$, i.e., a z component of **B** will mix X and Y, while an x component of **B** will mix Y and Z. The magnitude of the mixing will be determined by the magnetic energy $\sim g|\mu_b B|$ in relation to the exchange splitting For reference, at 700 mT, $\mu_b B$ is 40 μeV, which is not small relative to the singlet-triplet splitting $\hbar\omega_{st} = 126\ \mu$eV in orthorhombic $MAPbI_3$.

As an example, for a magnetic field applied along the [001] direction, taken as the **Z** direction, the magneto-exciton Hamiltonian, including exchange and the Zeeman effect, breaks down into two 2x2 submatrices as follows: The first submatrix describes coupling between D and Z states:

$$\widetilde{H}_{z,d}(B_{z)} = \begin{pmatrix} E_D & -B_z \frac{\mu_b}{2}(g_{e,z} - g_{h,z}) \\ -B_z \frac{\mu_b}{2}(g_{e,z} - g_{h,z}) & E_Z \end{pmatrix}. \tag{S43}$$

The energy eigenstates that are formed from the magnetically coupled Z and D levels have energies,

$$E_{zd}^{\pm} = \frac{1}{2} \left\{ E_D + E_Z \pm \sqrt{(E_{ZR} - E_D)^2 + \mu_b^2 B_z^2 (g_{e,z} - g_{h,z})^2} \right\} , \tag{S44}$$

and eigenvectors given by,

$$\psi_{zd}^{+} = \frac{1}{N_1} \begin{bmatrix} 1 \\ \frac{-B_z \mu_b (g_{e,z} - g_{h,z})}{2(E_1^{+} - E_z)} \end{bmatrix} \equiv \begin{bmatrix} \alpha_1 \\ -\beta_1 \end{bmatrix},$$

$$\psi_{zd}^{-} = \frac{1}{N_1}\begin{bmatrix} \frac{-B_z\mu_b\left(g_{e,z} - g_{h,z}\right)}{2\,(E_1^{-} - E_D)} \\ 1 \end{bmatrix} \equiv \begin{bmatrix} \beta_1 \\ \alpha_1 \end{bmatrix}. \tag{S45}$$

Here N_1 is a normalization factor and we note that α_1, β_1 are real. The other submatrix describes magnetic coupling of the *X* and *Y* states:

$$\widetilde{H}_{xy}(B_{z)} = \begin{pmatrix} E_X & -i\,B_z\frac{\mu_b}{2}\left(g_{e,z} + g_{h,z}\right) \\ +iB_z\frac{\mu_b}{2}\left(g_{e,z} + g_{h,z}\right) & E_Y \end{pmatrix} \tag{S46}$$

Solutions for the energies of the magnetically coupled X and Y states are,

$$E_{xy}^{\pm} = \frac{1}{2}\left\{E_X + E_Y \pm \sqrt{(E_X - E_Y)^2 + \mu_b^2 B_z^2\left(g_{e,z} + g_{h,z}\right)^2}\right\}, \tag{S47}$$

Corresponding to eigenvectors given by,

$$\psi_{xy}^{+} = \frac{1}{N_2}\begin{bmatrix} -i \\ \frac{B_z\mu_b\left(g_{e,z} + g_{h,z}\right)}{2(E_2^{+} - E_Y)} \end{bmatrix} \equiv \begin{bmatrix} -i\,\alpha_2 \\ \beta_2 \end{bmatrix},$$

$$\psi_{xy}^{-} = \frac{1}{N_2}\begin{bmatrix} \frac{-iB_z\mu_b\left(g_{e,z} + g_{h,z}\right)}{2(E_2^{-} - E_x)} \\ 1 \end{bmatrix} = \begin{bmatrix} i\;\beta_2 \\ \alpha_2 \end{bmatrix}. \tag{S48}$$

As above, N_2 is a normalization factor and we note that α_2, β_2 are real. It is important to note that as the magnetic energy increases, the triplet states *X* and *Y* mix, as do the *Z* and *D* states. The dipoles are given at zero magnetic field and at high magnetic field in Supplementary Table 2 and the energies are plotted as a function of the magnetic field in Supplementary Fig. 2.

Supplementary Table 2. Energies and transition dipoles for magneto excitons in a magnetic field oriented along the Z direction.

Transition dipoles are given at zero field and at non-zero field. The state index 1 to 4 is assigned to each level for convenience in the discussion of quantum beating of the magneto-exciton. **P** is the Kane matrix element.

State index	State index	Energy	Energy and Dipole at B_z=0	Dipole at $B_z \neq 0$
ψ_{xy}^{+}	1	E_{xy}^{+}	$E_X;\ \sqrt{2}\,P\mathcal{C}_X\hat{x}$	$\sqrt{2}\,P\left(i\,\alpha_2\mathcal{C}_x\hat{x} + \beta_2\mathcal{C}_y\hat{y}\right)$
ψ_{xy}^{-}	2	E_{xy}^{-}	$E_y;\ \sqrt{2}\,P\mathcal{C}_Y\hat{y}$	$\sqrt{2}\,P\left(-i\,\beta\mathcal{C}_x\,\hat{x} + \alpha_2\mathcal{C}_y\,\hat{y}\right)$
ψ_{zd}^{+}	3	E_{zd}^{+}	$E_D;\ 0$	$-\sqrt{2}\,P\beta_1\mathcal{C}_z\hat{z}$
ψ_{zd}^{-}	4	E_{zd}^{-}	$E_Z;\ \sqrt{2}\,P\mathcal{C}_z\hat{z}$	$\sqrt{2}\,P\alpha_1\mathcal{C}_z\hat{z}$

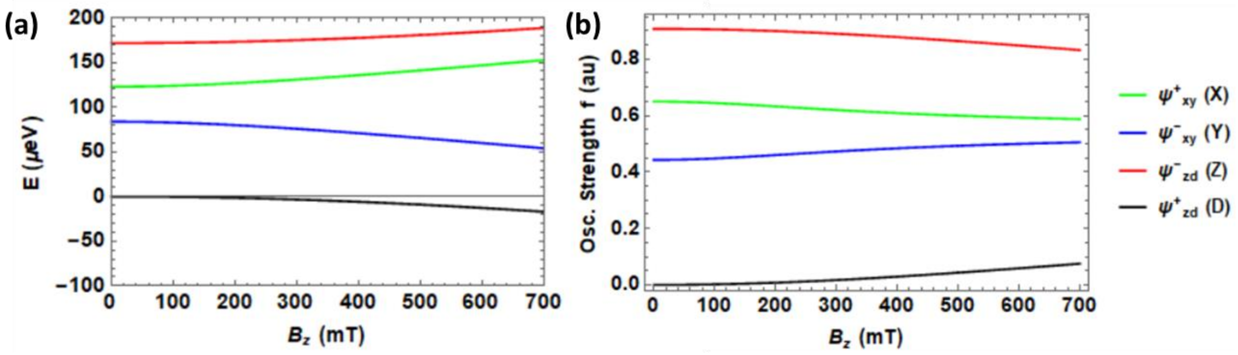

Supplementary Fig. 2. Energies and transition oscillator strengths for magneto-excitons in MAPbI3, B along [001]. Energies, panel (a) and oscillator strength, panel (b), are calculated with magnetic field applied along the [001] direction, taken as **z**. Lines are labelled according to the states in Supplementary Table 2**,** with the dipole character at zero magnetic field in parenthesis. For these calculations, $\hbar\omega_{st} = 126\ \mu$eV[6] ; Δ=1.4 eV[4]. The CF parameters $\delta = +349.8$ meV, ζ = +147.7 meV; $\kappa =$ 0.206 and the Kane energy, E_p=13.9 eV are determined by best fit to the measured electron and hole g-factors, resulting in $g^e_{[001]} = 2.52$, $g^h_{[001]} = -0.28$.

Section 2.2: Quantum beating model for magneto-excitons

In the transient photoinduced circular polarized reflection (c-PPR) quantum beating experiments described in the main text, a short optical pulse that is spectrally broad in comparison to the fine structure splitting is incident on the sample with wave vector **K** at time $t = 0$. With respect to the wave vector, the exciting pulse is arranged to be either left or right handed. For light propagating along the + **Z** direction, the left and right circular polarization vectors have positive and negative helicity, respectively:

$$\mathbf{e}_+ = 1/\sqrt{2}\ (\hat{\mathbf{x}} + i\,\hat{\mathbf{y}}), \qquad (left)$$

$$\mathbf{e}_- = 1/\sqrt{2}\ (\hat{\mathbf{x}} - i\,\hat{\mathbf{y}}), \qquad (right) \tag{S49}$$

We consider the wavevector **K** along the **X** direction for which,

$$\mathbf{e}_\pm(\mathbf{K} = K\hat{\mathbf{x}}) = \quad 1/\sqrt{2}\ (\pm i\,\hat{\mathbf{y}} - \hat{\mathbf{z}}) \tag{S50}$$

The magneto-exciton fine structure of the system consists of four states which we index, i, each with energy E_i and a transition dipole from the crystal ground state with is the vector $\mathbf{p}_\mathrm{i}$; these are given in .
Supplementary Table 2**.** When the exciting pulse arrives at time $t = 0$, an exciton is created by action of the electric field $\mathbf{e}_\pm$ on the crystal ground state $|G\rangle$. This state, $\Psi^\pm_{ex}$, at time $t = 0$ is given by[13],

$$|\Psi^\pm_{ex}\rangle = \mathbf{e}_\pm \cdot \mathbf{P}\,|G\rangle\ . \tag{S51}$$

We can represent this state as a superposition of the magneto-exciton fine structure levels ψ_i:

$$\Psi_{ex}^{\pm} = \sum_{i} (\mathbf{e}_{\pm} \cdot \mathbf{p_i})\, \psi_i \quad \equiv \sum_{i} A_i^{\pm}\, \psi_i \;;$$

$$A_i^{\pm} \equiv (\mathbf{e}_{\pm} \cdot \mathbf{p_i}) \tag{S52}$$

In this expression, the term $\mathbf{e}_{\pm} \cdot \mathbf{p_i}$ represents the relative strength of the dipole interaction of exciton level i with the light field, whose transition dipole is, $\mathbf{p_i} = \langle \psi_i | \mathbf{P} | G \rangle$, where G is the crystal ground state. These are given for each level in Supplementary Table 2. The exciton state subsequently evolves in time as,

$$\Psi_{ex}^{\pm}(t) = \sum_{i} A_i^{\pm}\, e^{-i\,t\frac{E_i}{\hbar}}\, \psi_i = \sum_{i} (\mathbf{e}_{\pm} \cdot \mathbf{p_i})\; e^{-i\,t\frac{E_i}{\hbar}}\, \psi_i \quad . \tag{S53}$$

In the c-PPR experiment, the system is interrogated by a + circularly polarized pulse, which we denote by $\mathbf{d}_{+} = 1/\sqrt{2}\ (+i\,\hat{\mathbf{y}} - \hat{\mathbf{z}})$, at a later time t. The signal, $S_{\pm}(t)$, corresponding to the state initially prepared by a pump pulse with polarization $\boldsymbol{e}_{\pm}$ is then proportional to,

$$S_{\pm}(t) \sim \left| \langle \Psi_{ex,d}^{+} | \Psi_{ex}^{\pm}(t) \rangle \right|^2 , \tag{S54}$$

where the state $\Psi_{ex,d}^{+}$ is,

$$\Psi_{ex,d}^{+} = \sum_{i} (\mathbf{d}_{+} \cdot \mathbf{p_i})\, \psi_i \;\; \equiv \sum_{i} B_i^{+}\, \psi_i \;\;;$$

$$B_i^{+} \equiv (\mathbf{d}_{+} \cdot \mathbf{p_i}) \tag{S55}$$

The measured signal, which we denote $QB(t)$, is the difference between the plus and minus signals:

$$\mathrm{QB(t)} \sim S_{+}(t) - S_{-}(t) \quad . \tag{S56}$$

Using the expressions worked out above and using the orthogonality of the states ψ_i we find,

$$S_{\pm}(t) \sim \left| \sum_{i} [B_i^{+}]^{*} A_i^{\pm} e^{-i\,t\frac{E_i}{\hbar}} \right|^2 , \tag{S57}$$

To determine the selection rules we will evaluate $S_{\pm}(t)$ using the definition,

$$D_i^{\pm}(t) = [B_i^{+}]^{*} A_i^{\pm}\; e^{-i\,t\frac{E_i}{\hbar}} \tag{S58}$$

The expression for the signal then becomes,

$$S_{\pm}(t) \sim \left| \sum_{i} D_i^{\pm}(t) \right|^2 . \tag{S59}$$

This can be expanded out:

$$S_{\pm}(t) \sim \sum_{i} \left| D_i^{\pm}(t) \right|^2 + \sum_{i \neq j} D_i^{\pm}(t)\; \left[D_j^{\pm}(t) \right]^{*} . \tag{S60}$$

The first term is constant in time and will not give rise to a beating signal. The second term is contains cross terms and is responsible for the beating. Discarding the time independent piece we write,

$$\delta_{\pm}(t) \sim \sum_{i \neq j} D_i^{\pm}(t)\ \left[D_j^{\pm}(t)\right]^* \quad . \tag{S61}$$

Then,

$$QB(t) \sim \delta_+(t) - \delta_-(t) \quad . \tag{S62}$$

We now consider the selection rules. In the general case, we find that all terms $D_i^{\pm}(t)$ are in general non-zero, and that six frequencies appear corresponding to the six possible energy differences between the four fine structure levels of the magneto-exciton. However, evaluating Eq. S62 for the special case of Voight geometry with applied magnetic field along the z-direction, and the light incident along a principal axis of the crystal (here taken as the x direction) we find two strong selection rules such that only four frequencies can appear in the quantum beating signature. To establish the selection rules explicitly we evaluate the terms $D_i^{\pm}(t)$ in Eq S58.

First selection rule: D/Z mixing: We first analyze the interference term corresponding to the pair of states $\psi_{zd}^{\pm}$ states which are magnetically coupled: Referring to Supplementary Table 2 we index these states as state 3, ψ_{zd}^{+}, and state 4, ψ_{zd}^{+}. Using $\mathbf{d}_+ = 1/\sqrt{2}\ \left(+i\,\hat{\mathbf{y}} - \hat{\mathbf{z}}\right)$ and referring to Supplementary Table 2 for the dipoles we find,

$$\begin{aligned} State\ 3:\quad D_3^{\pm} &= [B_3^{+}]^* A_3^{\pm}\, e^{-i\,t\frac{E_3}{\hbar}} = C_z^2\, \beta_1^2 P^2 e^{-i\,t\frac{E_3}{\hbar}} \quad , \\ State\ 4:\quad D_4^{\pm} &= [B_4^{+}]^* A_4^{\pm}\, e^{-i\,t\frac{E_4}{\hbar}} = C_z^2\, \alpha_1^2 P^2 e^{-i\,t\frac{E_4}{\hbar}} \quad . \end{aligned} \tag{S63}$$

We see that both terms are non-zero and the beating terms associated with right and left circular pump are equal. The corresponding quantum beat term associated with states 3, 4 is,

$$\delta_{\pm}^{3,4}(t) \sim D_3^{\pm}\left[D_{4\prime}^{\pm}\right]^* + D_4^{\pm}\left[D_3^{\pm}\right]^* = 2\, C_z^4\ \ \alpha_1^2\ \ \beta_1^2\ \ P^4 \cos\left(\frac{(E_3 - E_4)t}{\hbar}\right). \tag{S64}$$

Consequently in the quantum beating signal, Eq. S62, the interference terms involving states 3,4 cancel:

$$QB^{3,4}(t) = \delta_+^{3,4}(t) - \delta_-^{3,4}(t) = 0 \quad . \tag{S65}$$

We have arrived at the selection rule that the magnetically coupled $\psi_{zd}^{\pm}$ states cannot beat in the Voigt geometry, with the wave vector oriented along a principle symmetry axis of the crystal.

Second selection rule: X/Y mixing: We next analyze the beating associated with the magnetically coupled *X*,*Y* pair. For this case, again referring to .
Supplementary Table 2. Energies and transition dipoles for magneto excitons in a magnetic field oriented along **the** Z direction. and labelling ψ_{xy}^{+} as state 1 and ψ_{zd}^{-} as state 2, and using the dipoles listed in the table, we find,

$$State\ 1:\quad D_1^{\pm} = [B_1^{+}]^* A_1^{\pm}\, e^{-i\,t\frac{E_1}{\hbar}} = C_y^2\, \beta_2^2 P^2 e^{-i\,t\frac{E_1}{\hbar}} \quad ,$$

$$State\ 2: \quad D_2^{\pm} \ = \ [B_4^{+}]^* A_4^{\pm} \ e^{-i\,t\frac{E_4}{\hbar}} = \ C_y^2 \ \alpha_2^2 P^2 e^{-i\,t\frac{E_2}{\hbar}} \ . \tag{S66}$$

We again find the result that the beating terms associated with right and left circular pump are equal,

$$\delta_{\pm}^{1,2}(t)(t) \sim D_1^{\pm}\left[D_2^{\pm}\right]^* + \ D_2^{\pm}\left[D_1^{\pm}\right]^* = \ 2\,C_y^4 \quad \alpha_2^2 \quad \beta_2^2 \quad P^4 \cos\left(\frac{(E_1 - E_2)t}{\hbar}\right). \tag{S67}$$

Consequently in the quantum beating signal, Eq. S62, the state 1,2 interference terms cancel:

$$QB^{1,2}(t) \ = \delta_{+}^{1,2}(t) - \ \delta_{-}^{1,2}(t) \ = \ 0\,. \tag{S68}$$

We have arrived at the second selection rule, that the magnetically coupled $\psi_{xy}^{\pm}$ states cannot beat in the Voigt geometry, with the wave vector oriented along a principle symmetry axis of the crystal.

Analysis of the other four possible combinations shows that non-zero quantum beating signals are in possible with all four other combinations of states, with the exception of the case of zero applied magnetic field. At zero field, only one quantum beat frequency is possible for the light vector oriented along a principal axis of the crystal, as assumed above. This is because, i) all interferences involving the *D* state vanish due to the vanishing oscillator strength of the *D* state at zero field; ii), the *X/Y* mixing selection rule derived above leaves only two possible frequencies and iii), because bright excitons whose dipole is oriented along the direction of the wave vector, is oriented a principal axis of the crystal, cannot couple to the light, leaving a single possible frequency. Conversely, at finite magnetic field with the light wave vector oriented along a principal symmetry axis a total of four beat frequencies are possible. Then, for an arbitrary k-vector direction in Voight geometry, a total of six beat frequencies are possible at non-zero applied magnetic field, and two are allowed for zero applied field. The selection rules derived match those derived for time-resolved Faraday rotation in Voight geometry as developed in Ref. [13] and are confirmed by direct diagonalization of the magneto-exciton Hamiltonian and evaluation of the time dependent cPPR QB signal and its Fourier transform, shown in Supplementary Fig. 3 and Supplementary Fig. 4.

The calculations shown in these figures are made with the experimentally derived g-factors observed in the c-PPR quantum beating experiments described in the main text. The results show that if the quantum beating observed were due to excitons, there would be a clear beat signal at zero applied magnetic field, due to electron-hole-exchange related fine structure splitting. In Supplementary Fig. 3**,** which shows the calculated QB signal with applied magnetic field, **B** along [001] and incident light wavevector along [100], a principal axis, there is one allowed interference at zero field involving *Y-Z* fine structure levels. At high field, a clear signature associated with magnetic activation of the dark exciton oscillator strength would be observed, resulting in a total of four distinct beat frequencies, contrary to what is observed experimentally. In Supplementary Fig. 4. Calculated quantum beating signal for magneto-excitons in MAPbI3 for transient photoinduced circular polarized reflection, **B** along [-110].**,** calculated with applied magnetic field, **B** along [-110] and incident light wavevector along [110], there are two allowed interferences at

zero field while at high field, a total of six distinct beat frequencies become allowed due to magnetic activation of the "dark" exciton, again, contrary to what is observed experimentally.

We conclude from this analysis that the quantum beating signals experimentally observed in the c-PPR experiments are not due to excitons, but rather, are due either to the presence of resident carriers[10]; to formation of trions; or to resident carriers left behind after trion decay[14]. We turn in the next section to an analysis of quantum beating due to trions.

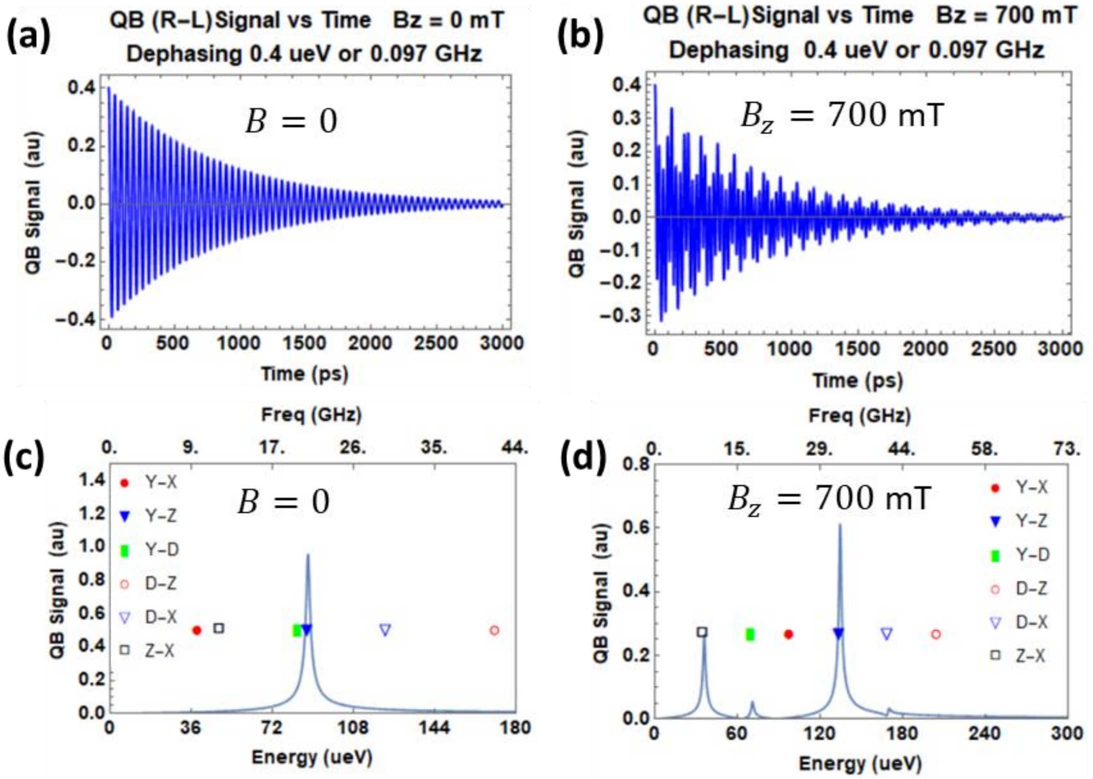

Supplementary Fig. 3. Calculated quantum beating signal for magneto-excitons in MAPbI3 for transient photoinduced circular polarized reflection, B along [001]. Quantum beating is calculated for Voigt geometry, with applied magnetic field, $\mathbf{B} = B_z\,\hat{\mathbf{z}}$ along [001] and incident light wavevector along [100] taken as $\hat{\mathbf{x}}$. The magneto-exciton fine structure and material parameters used in these simulations are shown in Supplementary Fig. 2. Panels (a, b) shows the time resolved c-PPR signal for zero magnetic field, panel (a), and an applied magnetic field of 700 mT, panel (b). Panels (c) and (d) show the Fourier transforms of the time-resolved quantum beating traces shown in panels (a) and (b) respectively. Beat signals are labelled based on the zero-field states to which the interfering magneto-exciton levels correspond, see Supplementary Fig. 2. For these calculations, damping of 0.4 μeV was assumed, consistent with the measured damping. Note that at zero field, there is one beat frequency corresponding to the interference between the *Y* and *Z* excitons; the *Z/X* exciton interference is forbidden with light wave vector along x, which cannot couple to the *X* state. All signals associated with the *D* state vanish at zero field due to the vanishing oscillator strength of this state at zero applied magnetic field. At non-zero magnetic field, a total of four beat frequencies are possible due to magnetic activation of the "*D*" state as described in the text.

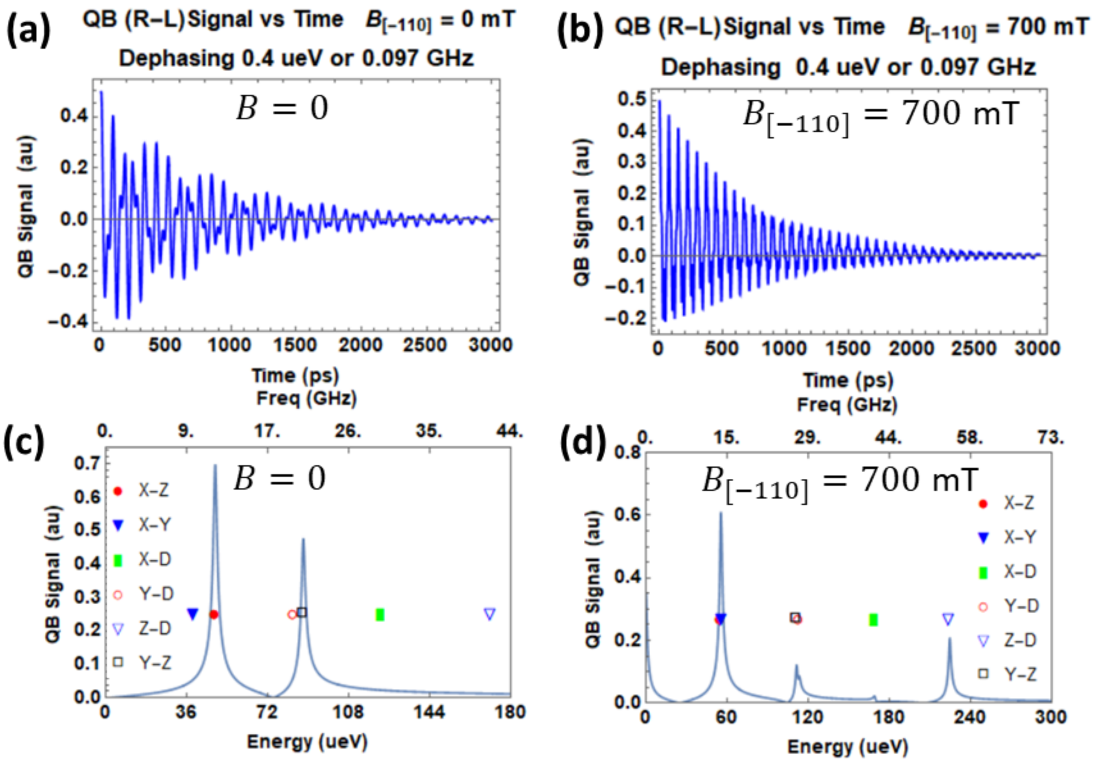

Supplementary Fig. 4. Calculated quantum beating signal for magneto-excitons in MAPbI3 for transient photoinduced circular polarized reflection, B along [-110].

Quantum beating is calculated for Voigt geometry, with applied magnetic field, **B** along [-110] and incident light wavevector along [110]. Panels (a, b) shows the calculated time resolved c-PPR signal for zero magnetic field, panel (a), and an applied magnetic field of 700 mT, panel (b). Panels (c) and (d) show the Fourier transforms of the time-resolved quantum beating traces shown in panels (a) and (b) respectively. Beat signals are labelled based on the zero-field states to which the interfering magneto-exciton levels correspond, see Supplementary Fig. 2. For these calculations, damping of 0.4 μeV was assumed, consistent with the measured damping. Note that at zero field, two beat frequencies emerge corresponding to the interferences between the *Y* and *Z* excitons and the *Z* and *X* excitons. All signals associated with the *D* state vanish at zero field due to the vanishing oscillator strength of this state at zero applied magnetic field. All six possible interferences are allowed for non-zero magnetic field since the light wave vector is not oriented along a principal axis of the crystal.

Supplementary Note 3: Trion magnetic field splitting and quantum beating

Here we analyze the level structure of trions in a magnetic field, the corresponding absorption spectra, and the expected quantum beating signature in the Voigt geometry. We show that a trion of a given charge type can produce one quantum beat frequency (during its lifetime). For trions with $\mathbf{B} = B_z\,\hat{\mathbf{z}}$, the beat frequency is $\mu_B\, g_{e,z} B_z$ for positive trions, or, $\mu_B\, g_{h,z} B_z$ for negative trions. This is in contrast with the result for resident electron or holes where there are two beat frequencies respectively corresponding to energies $\mu_B\, g_{e,z} B_z$ and $\mu_B g_{h,z}\, B_z$.

Trion creation- zero magnetic field

Supplementary Fig. 1 shows a conceptual schematic of the process of resonant creation of a positive trion in its ground state. The positive trion is visualized as two holes and one electron mutually bound. The electron representation is used for the conduction band (CB) and the hole-representation is used for the valence band (VB). Optical absorption promotes an electron from the VB into the conduction band. If the starting hole has spin up, this means that there is a missing electron with spin down. The photon promotes the remaining electron into the CB so that the final configuration comprises two spin-paired holes plus the electron in the CB. The bottom row in the figure shows the same process viewed in the electron representation. Notably, a positive trion in its lowest energy configuration can be viewed as a single electron excitation in the electron representation.

The matrix elements for creation of the trion are easiest to write in the electron representation. For the process shown in Supplementary Fig. 5, bottom row, the transition dipole matrix element between the initial state, i, and the final state, f, is, $\langle i|P|f\rangle = \langle v_i|\mathbf{P}|c_f\rangle$, where v_i represents the initial state of the valence band electron while c_f represents the final state of the conduction band electron. Unlike transitions involving the creation of free electron hole pairs or excitons, the initial state is not the crystal ground state, $|G\rangle$, but rather, the state of crystal with the initial charge carrier.

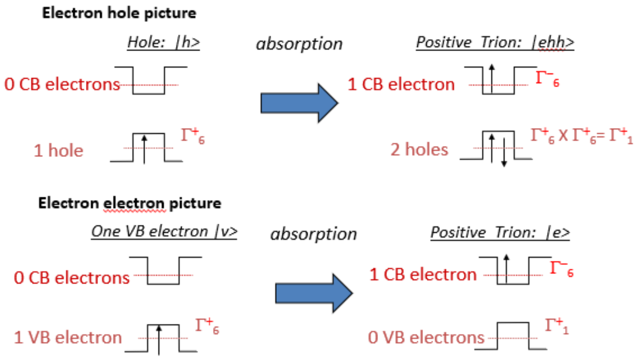

Supplementary Fig. 5. Creation of a positive trion by resonant optical absorption. System starts in the initial state with one hole, top left. Absorption of a photon leaves the system in the final state with 2 spin-paired holes and an electron, top right. Bottom pictures show the same process viewed in the electron representation for both bands. The irreducible representation labels follow the convention of KDWS[15] and for simplicity assume point symmetry $\boldsymbol{O_h}$corresponding to a cubic perovskite.

Trion has no fine structure in zero magnetic field

For perovskites of cubic symmetry (point group O_h), in zero magnetic field, electrons (holes) have symmetry $\Gamma_6{}^{-(+)}$ respectively, and are two-fold degenerate. Therefore, a positive/negative trion in its lowest energy configuration has symmetry $\Gamma_6{}^{-(+)}$ and is also two-fold degenerate. This follows since the two-like carriers are in a singlet configuration, i.e., $\Gamma_6{}^{-(+)} \otimes \Gamma_6{}^{-(+)} \rightarrow \Gamma_1{}^+$ for the singlet, and since $\Gamma_6{}^{-(+)} \otimes \Gamma_1{}^+ = \Gamma_6{}^{-(+)}$. Moreover, optical decay from a trion state to a single-carrier state is allowed for all polarizations: The x, y, and z components of the dipole operator all transform as $\Gamma_4{}^-$, since $\Gamma_4{}^- \otimes \Gamma_6{}^{-(+)}$contains $\Gamma_6{}^{-(+)}$. Furthermore, the matrix elements are equal for the x, y, and z components by symmetry. This argument is easily generalized to the tetragonal and

orthorhombic cases[2]. For the orthorhombic case, electrons (holes) have symmetry ${\Gamma_5}^{-(+)}$; therefore, a positive/negative trion has symmetry ${\Gamma_5}^{-(+)}$ and is also two-fold degenerate.

Consequently the ground state of the trion in zero magnetic field has no fine structure splitting. This is well known in the literature, see for example [16,17].

Section 3.1 Magnetic field splitting – Zeeman model for trion

The Zeeman Hamiltonian for the positive trion can be written for cubic symmetry by summing the Zeeman terms of the three particles:

$$H_M = g_e\mu_B \mathbf{J_e}\cdot\mathbf{B} + g_h\mu_B \mathbf{J_{h,1}}\cdot\mathbf{B} + g_h\mu_B \mathbf{J_{h,2}}\cdot\mathbf{B} \quad , \tag{S69}$$

where, $\mathbf{J_e}, \mathbf{J_h}$ are the vector total angular momentum operators for the electron and hole, respectively, μ_B is the Bohr magneton, and g_e, g_h are the electron and hole g-factors. For orthorhombic symmetry, this becomes,

$$H_M = \mu_B\left\{\left(g_{e,x}J_{\boldsymbol{e},\boldsymbol{x}} + g_{h,x}(J_{h1,x} + J_{h2,x})\right)B_x + \left(g_{e,y}J_{\boldsymbol{e},\boldsymbol{y}} + g_{h,y}(J_{h1,y} + J_{h2,y})\right)B_y + \left(g_{e,z}J_{e,z} + g_{h,z}(J_{h1,z} + J_{h2,z})\right)B_z\right\}. \tag{S70}$$

In the electron/hole representation, we write the trion state as a direct product of the three carrier wavefunctions. Consider a positive trion:

$$|ehh\rangle = |e\rangle|h_1\rangle|h_2\rangle \quad . \tag{S71}$$

Then the Zeeman splitting of the positive trion in magnetic field field $\mathbf{B}$ is, for cubic symmetry,

$$\langle ehh|H_m|ehh\rangle = g_e\mu_B\langle e|\mathbf{J_e}|e\rangle\cdot\mathbf{B} + g_h\mu_B\ \left(\langle h_1|\mathbf{J_{h1}}|h_1\rangle + \langle h_2|\mathbf{J_{h2}}|h_2\rangle\right)\cdot\mathbf{B} \quad . \tag{S72}$$

The generalization for orthorhombic symmetry is,

$$\langle ehh|H_m|ehh\rangle = \sum_{i=x,y,z}\left\{g_{e,i}\mu_b J_{e,i} + g_{h,1}\mu_b\left(J_{h1,i} + J_{h2,i}\right)\right\}B_i. \tag{S73}$$

For $\mathbf{B} = B_z\,\hat{\mathbf{z}}$, this yields, for the positive trion,

$$\langle ehh|H_m|ehh\rangle = \pm g_{ez}\mu_B\,\frac{B_z}{2}. \tag{S74}$$

Parallel arguments lead to the conclusion that, a negative trion has energy,

$$\langle eeh|H_m|eeh\rangle = \pm g_{hz}\mu_B\,\frac{B_z}{2}. \tag{S75}$$

These results are summarized schematically in Supplementary Fig. 6. A positive trion in a magnetic field will split into two lines separated in energy by $g_{e,z}\mu_B\,B_z$, while the splitting for a negative trion is $g_{h,z}\mu_B\,B_z$; these energy separations correspond to the Larmor precession frequencies observed in quantum beating experiments.

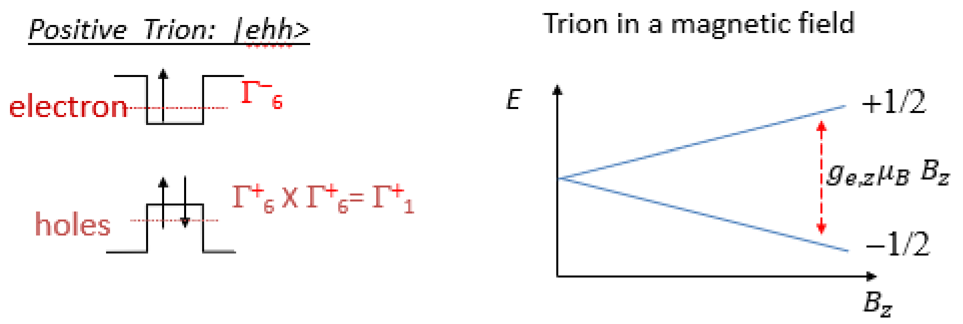

Supplementary Fig. 6. Positive trion level splitting in a magnetic field. Schematic that shows positive trion, $|\boldsymbol{ehh}\rangle$, left. The Zeeman splitting is depicted schematically on the right. The splitting for a positive trion is $\boldsymbol{g_{e,z}\mu_B\ B_z}$. The splitting for a negative trion is $\boldsymbol{g_{h,z}\mu_B\ B_z}$.

Section 3.1: Quantum beating signature of trion

Now we analyze the quantum beating signature of the trion in Voigt geometry. For definiteness we will discuss only the positive trion, and analyze the system for specificity with the applied magnetic field along z, with light with wave-vector $\mathbf{K} = K\,\hat{\mathbf{x}}$, with associated polarization vectors $\mathbf{e}_\pm$ given in Eq. S50. We consider a short pulse at t=0 takes the system from initial state $|v_1\rangle$, for specificity, to a trion state $|\Psi^{\pm}_{t,1}\rangle$. This state at time $t = 0$ is given by[13],

$$|\Psi^{\pm}_{t,1}\rangle \sim \mathbf{e}_\pm \cdot \mathbf{P}\,|v_1\rangle\ , \tag{S76}$$

where **P** is the momentum operator. This state can be represented as a coherent superposition of the two trion sublevels $|c_1\rangle$ and $|c_2\rangle$, with amplitudes $C_1^{\pm}$ and $C_2^{\pm}$, respectively. The amplitudes at t = 0 are proportional to $\mathbf{e}_\pm \cdot \langle c_1|\mathbf{P}|v_1\rangle$ and $\mathbf{e}_\pm \cdot \langle c_2|\mathbf{P}|v_1\rangle$:

$$C_1^{\pm} = (\,\mathbf{e}_\pm \cdot \langle c_1|\mathbf{P}|v_1\rangle) = P\ \mathcal{C}_Z/\sqrt{2}\ ,$$

$$C_2^{\pm} = (\,\mathbf{e}_\pm \cdot \langle c_2|\mathbf{P}|v_1\rangle) = \pm P\,\mathcal{C}_Y/\sqrt{2}\ . \tag{S77}$$

Here, P is the Kane momentum matrix element and we used the Bloch functions for the orthorhombic phase, given for the valence and conduction bands in Eqs. S2 and Eq. S12 above. After time t has elapsed, the trion superposition state evolves due to the different energies of the trion with spin up versus spin down:

$$C_1^{\pm}(t) = C_1^{\pm}\,e^{-i\,t\frac{E_{c1}}{\hbar}}\ ; \qquad C_2^{\pm}(t) = C_2^{\pm}\,e^{-i\,t\frac{E_{c2}}{\hbar}}\ . \tag{S78}$$

Consequently the evolution of the trion state at time t is given by,

$$|\Psi^{\pm}_{t1}(t)\rangle \sim C_1^{\pm}(t)|c_1\rangle + C_2^{\pm}(t)\ |c_2\rangle\ . \tag{S79}$$

In the c-PPR experiment, the system is interrogated by a + circularly polarized pulse, which we denote by $\mathbf{d}_+ = 1/\sqrt{2}\ (+i\,\hat{\mathbf{y}} - \hat{\mathbf{z}})$, arriving at a later time t. The signal, $S_\pm(t)$, corresponding to the state initially prepared by a pump pulse with polarization $\mathbf{e}_\pm$ is proportional to,

$$S_\pm(t) \sim \left|\langle \Psi^{+}_{t1,d}|\Psi^{\pm}_{t1}(t)\rangle\right|^2\ , \tag{S80}$$

where the state $\Psi^{+}_{t,d}$ is,

$$|\Psi_{t1,d}^{+}\rangle \sim \mathbf{d}_{+} \cdot \mathbf{P}|v_1\rangle \;,$$

$$|\Psi_{t1,d}^{\pm}\rangle \sim C_1^{+}|c_1\rangle + C_2^{+}|c_2\rangle \;. \tag{S81}$$

The measured signal, which we denote $QB(t)$, is the difference between the plus and minus signals $QB(t) \sim S_{+}(t) - S_{-}(t)$.

Using the expressions worked out above and using the orthogonality of the states $|c_i\rangle$ we find,

$$S_{\pm}(t) \sim \left|\sum_{i=1}^{2}[C_i^{+}]^{*}C_i^{\pm}e^{-i\,t\frac{E_{ci}}{\hbar}}\right|^{2} \;. \tag{S82}$$

Substituting in the expressions in Eq. S77 and evaluating, we find the QB signal as,

$$QB(t) \sim P^4\, \mathcal{C}_Y^2\mathcal{C}_Z^2\, \cos\big(g_{e,z}\mu_B\, B_z t\big)\,, \tag{S83}$$

where we used $E_{c1} - E_{c2} = g_{e,z}\mu_B\, B_z$. Thus we see that the QB signal for the positive trion is determined by the Zeeman splitting which is governed by the electron g-factor.

Identical arguments apply in considering trion formation from initial state $|v_2\rangle$. Here, absorption of a photon with polarization $\mathbf{e}_{\pm}$ leads to trion state $|\Psi_{t,2}^{\pm}\rangle$. This state at time $t = 0$ is given by[13] ,

$$|\Psi_{t,2}^{\pm}\rangle \sim \mathbf{e}_{\pm} \cdot \mathbf{P}|v_2\rangle \;, \tag{S84}$$

This state can again be represented as a coherent superposition of the two trion sublevels $|c_1\rangle$ and $|c_2\rangle$, with amplitudes $D_1^{\pm}$ and $D_2^{\pm}$, respectively. The amplitudes at $t = 0$ are proportional to $\mathbf{e}_{\pm} \cdot \langle c_1|\mathbf{P}|v_2\rangle$ and $\mathbf{e}_{\pm} \cdot \langle c_2|\mathbf{P}|v_2\rangle$:

$$D_1^{\pm} = (\,\mathbf{e}_{\pm} \cdot \langle c_1|\mathbf{P}|v_2\rangle) = \mp P\, \mathcal{C}_Y/\sqrt{2} \;,$$

$$D_2^{\pm} = (\,\mathbf{e}_{\pm} \cdot \langle c_2|\mathbf{P}|v_2\rangle) = -P\, \mathcal{C}_Z/\sqrt{2} \;. \tag{S85}$$

The state evolves as before in time:

$$|\Psi_{t2}^{\pm}(t)\rangle \sim D_1^{\pm}(t)|c_1\rangle + D_2^{\pm}(t)\,|c_2\rangle \;. \tag{S86}$$

The signal, $S_{\pm}(t)$ in this case is proportional to,

$$S_{\pm}(t) \sim \left|\langle \Psi_{t2,d}^{+}|\Psi_{t2}^{\pm}(t)\rangle\right|^{2}, \tag{S87}$$

where the state $\Psi_{t2,d}^{+}$ is,

$$|\Psi_{t2,d}^{+}\rangle \sim \mathbf{d}_{+} \cdot \mathbf{P}|v_2\rangle \;,$$

$$|\Psi_{t2,d}^{\pm}\rangle \sim D_1^{+}|c_1\rangle + D_2^{+}|c_2\rangle \;. \tag{S88}$$

The measured QB signal $QB(t) \sim S_{+}(t) - S_{-}(t)$ evaluates to,

$$QB(t) \sim P^4\, \mathcal{C}_Y^2\mathcal{C}_Z^2\, \cos\big(g_{e,z}\mu_B\, B_z t\big)\,, \tag{S89}$$

Identical to what we found for the $|v_1\rangle$ initial state.

Supplementary Note 4: Supplementary data figures.

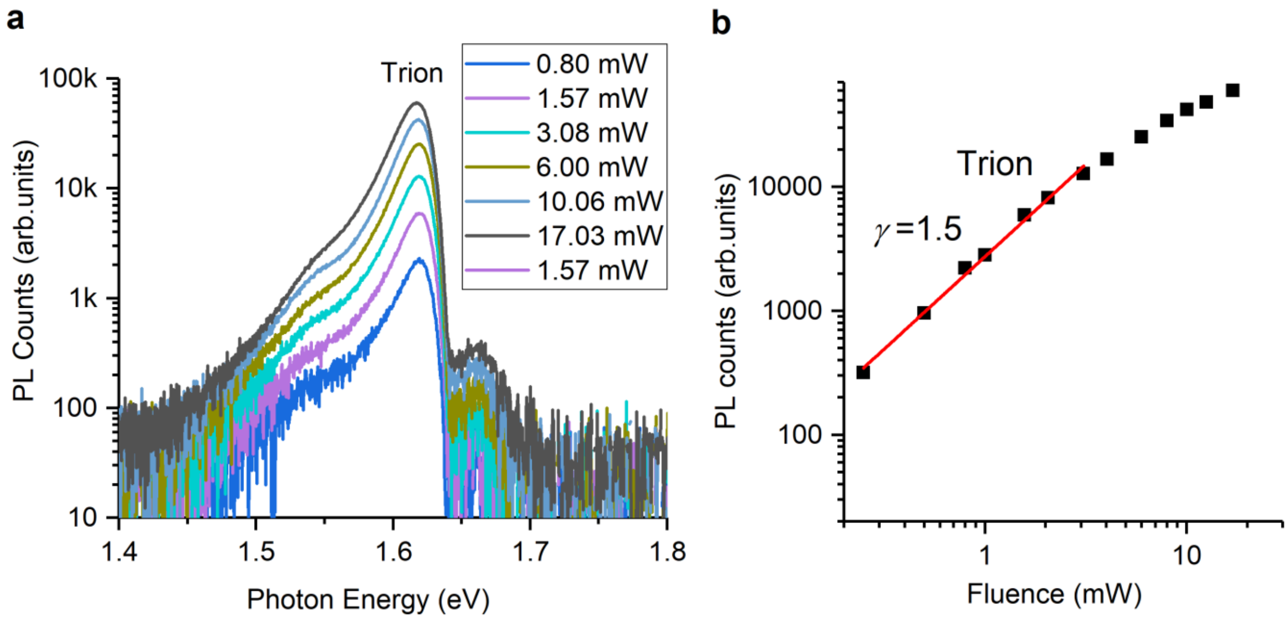

Supplementary Fig. 7. Photoluminescence spectra of a MAPbI3 crystal measured at 10K as a function of pump fluence. a PL spectra at different pump fluence that shows a dominant trion band ~ 1.62eV. **b** Plot of trion PL peak strength versus the pump fluence in log scale. The red line is a linear fitting with the slope $\gamma = 1.5$.

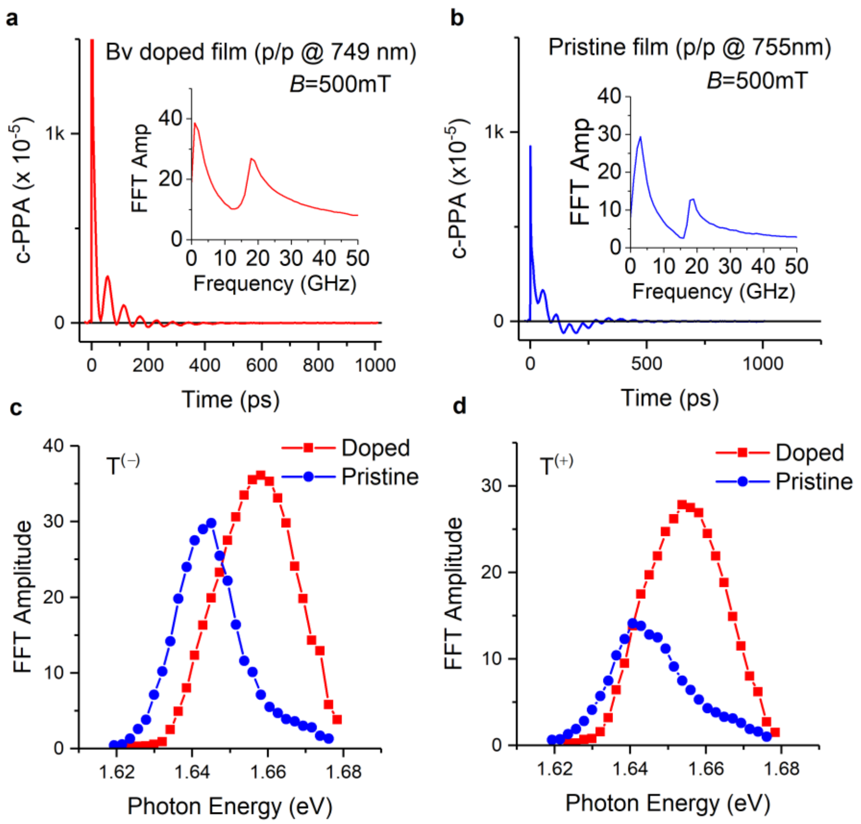

Supplementary Fig. 8. Comparison between photoinduced QBs in 'pristine' (that is in fact slightly p-type) and 'compensated' $MAPbI_3$ films. Panels a and b show the QBs measured by circular photoinduced absorption (c-PPA(t)); the insets show the corresponding FFT of the QB oscillations that contain two different QB components which we identify as due to photogenerated $T^{(+)}$ and $T^{(-)}$, respectively. Panels c and d show the excitation spectra of the FFT amplitude for the two trion, respectively. Here blue circles are for the 'pristine' film and red circles are for the 'compensated' film.

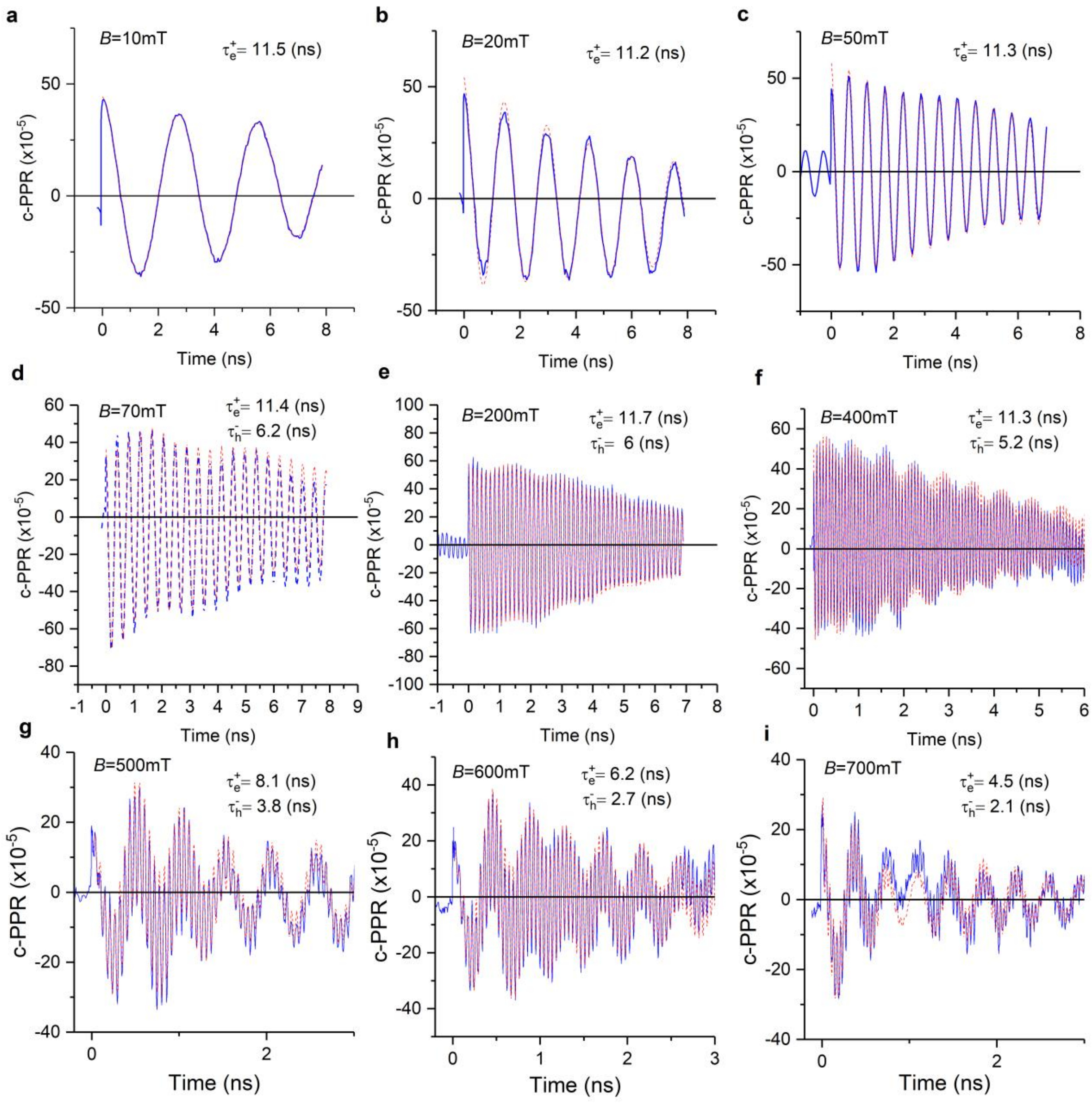

Supplementary Fig. 9. The c-PPR(t) dynamics at various *B*'s and extracted spin relaxation time of the positive (fast oscillation) and negative (slow oscillation) trions measured along [100] with B directed along [001]. The red dashed lines are fittings using equation (2) in the main text. The extracted spin relaxation times are denoted.

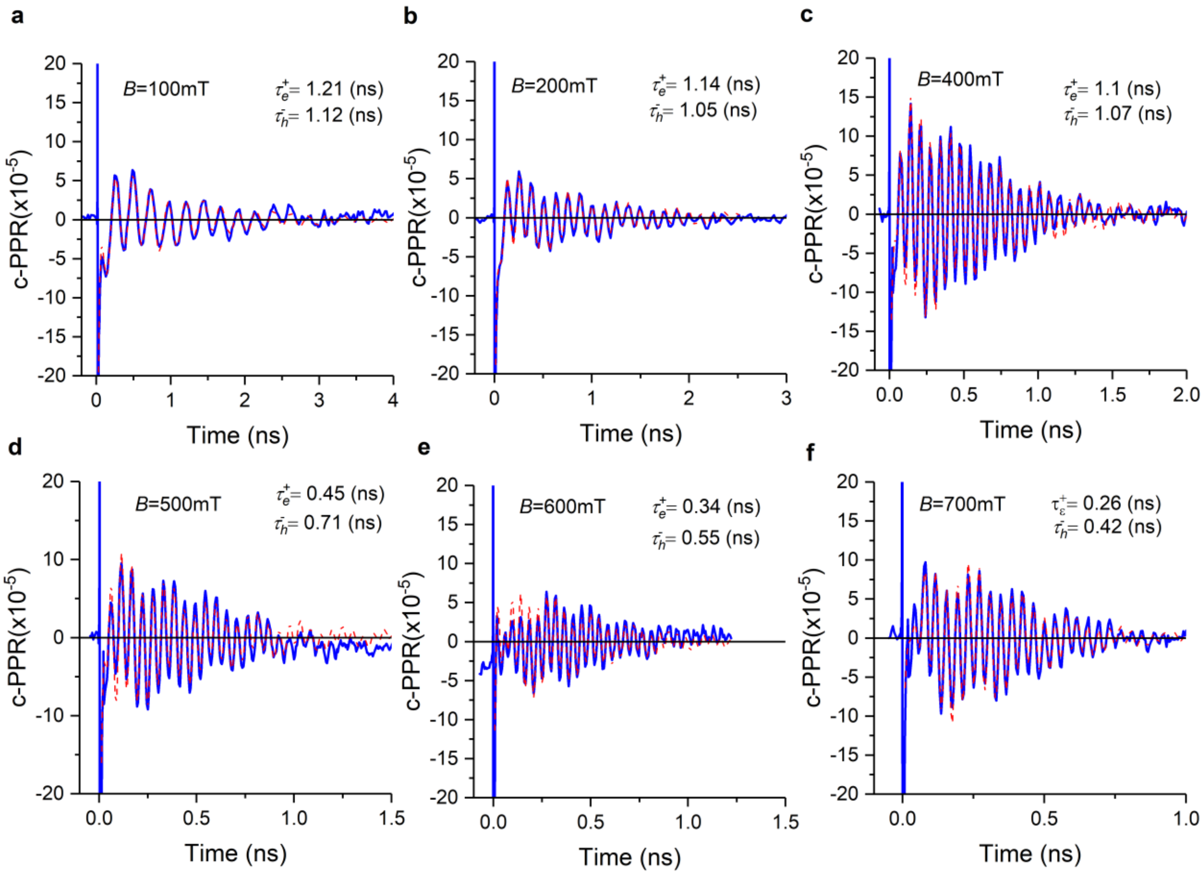

Supplementary Fig. 10. The c-PPR(t) dynamics at various *B*'s and extracted spin relaxation time of the positive (fast oscillation) and negative (slow oscillation) trions measured along [110] with B directed along [1$\bar{1}$0]. The red dashed lines are fittings using equation (2) in the main text. The extracted spin relaxation times for the two trions are denoted.

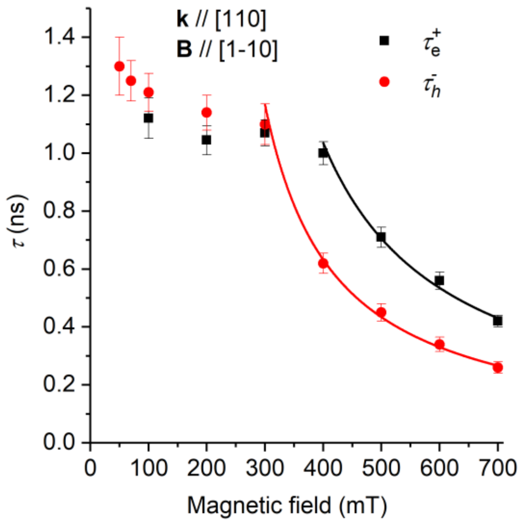

Supplementary Fig. 11. The spin relaxation time, τ of positive and negative trions (τ_e^+ and τ_h^-, respectively) measured at 4K with k along [110] with B directed along [1$\bar{1}$0]. Error bars derived from the least mean square fit of the c-PPR dynamics in Fig. 3 and Supplementary Fig. 10 using Eq. (2) in the main text. The lines through the data points for B > 400 mT are fits using a model described in the text where $\tau \sim 1/B$. The longest τ is about 1.2 ns, which is much shorter than the pulse to pulse time interval of ~ 12.5 ns.

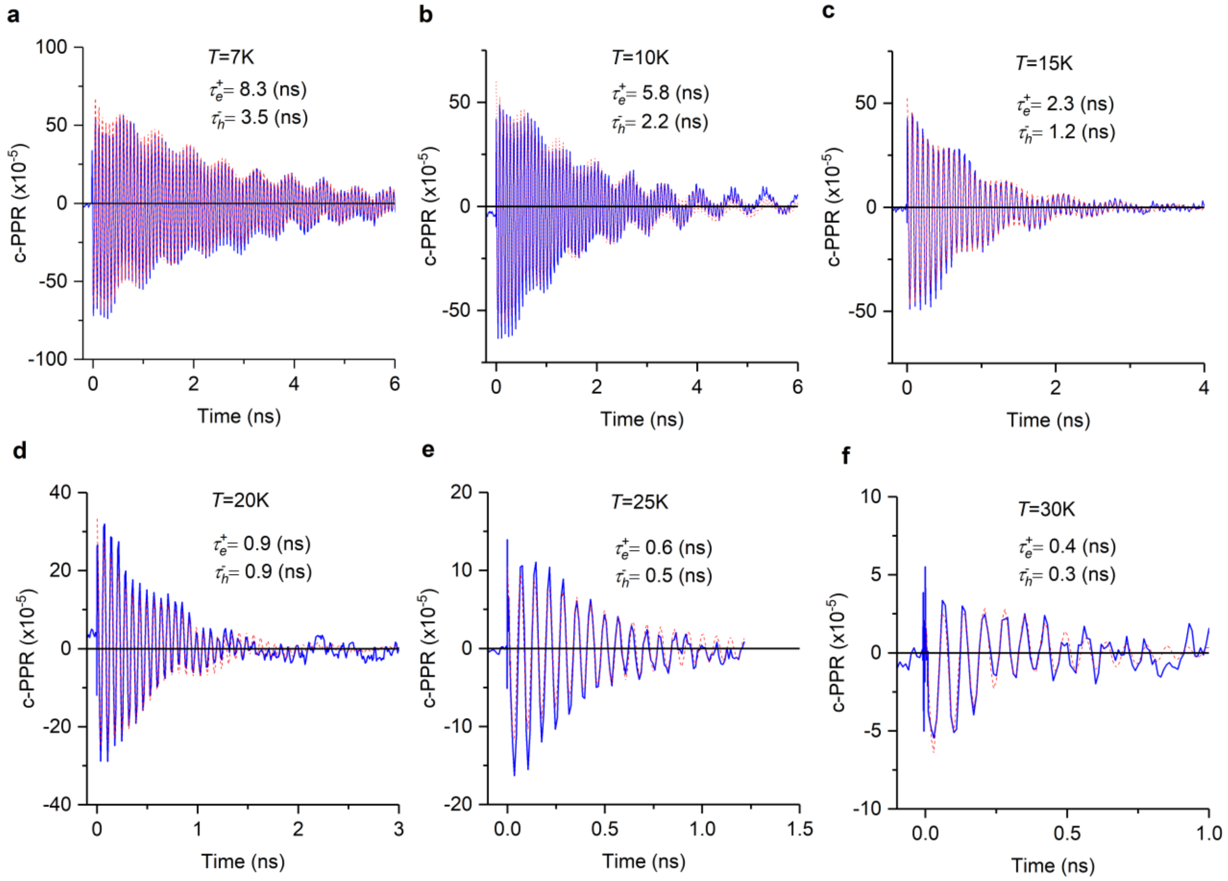

Supplementary Fig. 12. Temperature dependence of the c-PPR(t) response dynamics and the extracted spin lifetime of positive (τ_e^+) and negative (τ_h^-) trions measured along [100] with *B* = 400 mT directed along [001]. The red dashed lines are fits using equation (2) in the main text. The extracted spin relaxation times for the two trions are denoted.

Supplementary References

1. Even, J., Pedesseau, L., Jancu, J.-M. & Katan, C. Importance of spin−orbit coupling in hybrid organic/inorganic perovskites for photovoltaic applications. *J. Phys. Chem. Lett.* **4**, 2999 -3005 (2013).
2. Sercel, P.C., Lyons, J. L., Bernstein, N. & Efros, A. L. Quasicubic model for metal halide perovskite nanocrystals. *J. Chem. Phys.* **151**, 234106 (2019).
3. Nagamune, Y., Takeyama, S. & Miura, N. Exciton spectra and anisotropic Zeeman effect in PbI2 at high magnetic fields up to 40 *T. Phys. Rev. B* **43**, 12401 (1991).
4. Yu, Z. G. Effective-mass model and magneto-optical properties in hybrid perovskites. *Sci. Rep.* **6**, 28576 (2016).
5. Geng, W., Zhang, L., Zhang, Y.-N., Lau, W.-M. & Liu, L.-M. First-Principles Study of Lead Iodide Perovskite Tetragonal and Orthorhombic Phases for Photovoltaic. *J. Phys. Chem. C* **118**, 19565−19571(2014).
6. Ben Aich, R., Ben Radhia, S., Boujdaria, K., Chamarro, M. & Testelin, C. Multiband K·P model for tetragonal crystals: Application to hybrid halide perovskite nanocrystals. *J. Phys. Chem. Lett.* **11**, 808−817, (2020).
7. Kane, E.O. Band structure of Indium Antimonide. *J. Phys. Chem. Solids* **1**, 249-261 (1957).
8. Luttinger, J. M. Quantum theory of cyclotron resonance in semiconductors: General Theory. *Phys. Rev.* **102**, 1030-1041 (1956).
9. Lӧwdin, P.-O. A note on the quantum mechanical perturbation theory. *J. Chem. Phys.* **19**, 1396-1401 (1951).
10. Belykh, V.V. et al. Coherent spin dynamics of electrons and holes in$CsPbBr_3$ perovskite crystals. *Nat. Comm.* **10**, 673 (2019).
11. Sercel, P.C. et al. Exciton fine structure in perovskite nanocrystals. *Nano Lett.* **19**, 4068-4077 (2019).
12. Sercel, P. C., Vardeny, Z. V.& Efros, A. L. Circular dichroism in non-chiral metal halide perovskites. *Nanoscale* **12**, 18067–18078 (2020).
13. Odenthal, P. et al. Spin-polarized exciton quantum beating in hybrid organic–inorganic perovskites. *Nature Physics* **13**, 894 (2017).
14. Glazov M. M. Coherent Spin Dynamics of Electrons and Excitons in Nanostructures (A Review). *Phys. Solid State* **54**, 1–27 (2012).
15. Koster, G. F., Dimmock, J. O., Wheeler, R. G.& Statz, H. *Properties of the Thirty-Two Point Groups* (MIT Press, Cambridge, 1963).
16. Bayer, M. et al. Fine structure of neutral and charged excitons in self-assembled In(Ga)As/(Al)GaAs quantum dots. *Physical Review B* **65**, 195315-195337 (2002).
17. Dyakonov, M. I., (Ed.) Spin Physics in Semiconductors (Springer International Publishing AG, 2017).
